# Next-generation probiotics and engineered BEVs for precision therapeutics in osteoporosis

**DOI:** 10.3389/fnut.2025.1581971

**Published:** 2025-07-01

**Authors:** Jiecheng Wei, Wenshuo Ding, Kaiyi Song, Yongkang Zhang, Qi Luo, Chan Qi

**Affiliations:** ^1^Queen Mary School, Jiangxi Medical College, Nanchang University, Nanchang, China; ^2^The First Affiliated Hospital of Nanchang University, Nanchang, China; ^3^Department of Pulmonary and Critical Care Medicine, The Sixth Medical Center of Chinese PLA General Hospital, Beijing, China; ^4^School of Basic Medical Sciences, Jiangxi Medical College, Nanchang University, Nanchang, China; ^5^The First Hospital of Nanchang, The Third Affiliated Hospital of Nanchang University, Nanchang, China

**Keywords:** osteoporosis, probiotics, gut microbiota, bone health, inflammation, next-generation probiotics, microbial-targeted therapeutics, bacterial extracellular vesicles

## Abstract

Osteoporosis, characterized by reduced bone density and increased fracture risk, faces limitations with conventional therapies due to adverse effects and poor gut microbiota modulation. Emerging strategies leveraging probiotics and bacterial extracellular vesicles (BEVs) offer novel therapeutic potential by targeting the gut-bone axis. Engineered probiotics and next-generation formulations enhance osteoprotection via immunomodulation, metabolite production (e.g., SCFAs), and neuroendocrine regulation. BEVs, as biocompatible nanocarriers, enable targeted delivery of osteogenic factors while circumventing colonization challenges. Synthetic biology advances facilitate precision engineering of probiotics and BEVs, improving therapeutic efficacy and scalability. This review highlights pre-clinical and clinical progress, challenges in standardization and safety, and future directions for microbiome-based interventions to revolutionize osteoporosis management. Integrating engineered probiotics with BEV technology promises transformative approaches for bone health restoration.

## 1 Introduction

Osteoporosis (OP) is a chronic bone disease that is characterized by a decrease in bone mineral density and a deterioration in bone structure, leading to an increased risk of fractures, especially in postmenopausal women (1). While conventional treatments, including bisphosphonates, selective estrogen receptor modulators (SERMs), and calcium and vitamin D supplementation, have been shown to effectively reduce fracture risk, they are often associated with adverse side effects, such as gastrointestinal disturbances, atypical fractures, and osteonecrosis of the jaw (2, 3). Moreover, these treatments do not address the gut dysbiosis commonly observed in individuals with osteoporosis (4–6). This highlights the urgent need for the accelerated development of novel pharmacological interventions that minimize side effects. Such advancements have the potential to reshape the treatment landscape for osteoporosis in the near future.

Live beneficial bacteria, known as probiotics, are often used as a complementary therapy (7). Over 1,000 clinical trials have investigated probiotics for diverse pathologies, including gastrointestinal, metabolic and chronic diseases (colorectal cancer (8), multiple sclerosis (9); rheumatoid arthritis (10)), with mixed therapeutic outcomes. Currently, probiotics such as *Lactobacillus reuteri* (11), *Lactobacillus paracasei* (12), *Bifidobacterium longum* (13) and *Akkermansia muciniphila* (AKK) (14) have been shown to be potential targets for the treatment of osteoporosis. While promising, clinical efficacy is often constrained by poor gut colonization, inter-strain variability, dosage inconsistencies, and dynamic host-microbe interactions (15). To address these limitations, synthetic biology has emerged as a pivotal tool for engineering probiotics with enhanced therapeutic precision and functionality.

Next-Generation Probiotics (NGPs) leverage synthetic circuits to sense microenvironmental cues and dynamically deliver therapeutic payloads, such as enzymes for metabolic disorders (e.g., phenylketonuria, hyperammonemia) or anti-inflammatory molecules (16–18). These engineered systems also serve as *in situ* diagnostics, secreting therapeutic proteins in response to disease biomarkers (19).

Bacterial-derived extracellular vesicles (BEVs), phospholipid bilayer nanostructures measuring 40−200 nm in diameter (20), represent emerging nanoscale delivery platforms in biomedicine due to their tiny structure, mild toxicity and good biocompatibility (21). They are engineered to target bone tissue and can carry a variety of substances, including miRNAs, DNA, proteins, cytokines and other factors that regulate the progression of osteoporosis (22). In contrast to the low productivity of mammalian EVs (MEVs), BEVs derived from high-density batch-cultured probiotics possess a rapid proliferative capacity, allowing them to be mass-produced and tailored to synthetic biology (23). Particularly within the gut-bone regulatory network, BEV-mediated therapeutic strategies show remarkable capacity to modulate the onset and progression of OP.

We summarize the progress of both key common and rare probiotics in pre-clinical and clinical studies related to osteoporosis and explore the potential clinical applications of the latest engineering toolbox for bone health.

## 2 Osteoporosis

Osteoporotic fractures, a hallmark of systemic skeletal fragility, disproportionately affect the hip and vertebrae but impose the greatest socioeconomic burden through non-hip, non-vertebral fractures, which account for the majority of incident cases (8, 9). Hip fractures—characterized by acute pain, immobility, and high short-term mortality—pre-dominantly occur in women over 80 years, with global incidence showing marked geographical disparities (> 10-fold variation) and an estimated 2.7 million cases in 2010, half of which were potentially preventable through osteoporosis mitigation (10, 20, 21). Vertebral fractures, the most prevalent osteoporotic fractures, often evade clinical detection yet serve as critical predictors of subsequent fragility fractures, including hip fractures (14–16). While their heterogeneous presentation (ranging from asymptomatic to debilitating) complicates epidemiological analysis, standardized diagnostic criteria are emerging to refine their classification and clinical relevance (18, 19).

Notably, secular trends in fracture epidemiology reveal diverging patterns: Hip fracture rates have declined in North America but risen in Asia, whereas non-hip fractures exhibit less consistent trajectories (21–23). These shifts likely reflect complex interactions between lifestyle changes, urbanization, obesity trends, and screening practices. Despite advances in understanding, unmet needs persist in fracture prevention and global equity in osteoporosis care, particularly given the substantial morbidity, mortality, and economic costs linked to underdiagnosed vertebral fractures (13, 17).

### 2.1 Bone remodeling and age-related pathophysiology

The adult skeleton comprises cortical and trabecular bone, with site-specific pre-dominance: Vertebrae are rich in trabecular bone, while long bones primarily consist of cortical bone. Bone remodeling—a tightly coupled process of resorption and formation—occurs in discrete remodeling units. Orchestrated by osteoclast-mediated resorption followed by osteoblast-driven formation, this cycle renews the skeleton over ∼10 years while maintaining equilibrium in healthy adults (24, 25) In contrast, bone modeling, pre-dominant during skeletal development, decouples resorption and formation to optimize bone geometry in response to mechanical stress, persisting in adulthood under loading conditions (26, 27).

Genetic factors account for 50–85% of bone mineral density (BMD) variance, with genome-wide association studies (GWAS) identifying > 100 loci linked to bone strength and fracture risk. While most loci exert small polygenic effects, monogenic disorders have elucidated critical pathways (e.g., RANK/RANKL/OPG, Wnt signaling) governing bone remodeling and structural integrity (28).

### 2.2 Effects of osteoporosis drugs on bone remodeling and modeling

Osteoporosis drugs exert their effects on bone through distinct mechanisms, targeting either bone resorption or formation. Antiresorptive drugs primarily inhibit osteoclast recruitment and activity, reducing the rate of bone remodeling and allowing for a modest increase in bone mineral density (BMD) (29). By decreasing the number of remodeling units, these drugs reduce the negative remodeling balance, leading to increased secondary mineralization and preservation of bone mass and structure.

Denosumab, a monoclonal antibody targeting receptor activator of nuclear factor κB ligand (RANKL), has shown particular efficacy in improving cortical bone structure by increasing thickness and decreasing porosity. This effect may be due to its enhanced pharmacokinetic properties compared to bisphosphonates, allowing better accessibility to cortical bone (30).

Anabolic drugs stimulate bone formation through both remodeling and modeling processes. Teriparatide, a recombinant parathyroid hormone, promotes modeling-based bone formation on cancellous, endosteal, and periosteal surfaces, particularly in the early stages of treatment. However, the majority of its anabolic effects in cancellous bone are achieved through remodeling with overfilling of remodeling units (31, 32). In cortical bone, treatment with teriparatide may initially increase total bone area and cortical porosity, forming hypomineralized new bone. Despite these early changes, long-term treatment has been associated with increased bone strength and localized cortical thickness at mechanically loaded sites (33, 34).

Romosozumab, a monoclonal antibody targeting sclerostin, an osteocyte-derived inhibitor of bone formation, exhibits unique anabolic effects. Early treatment with romosozumab induces large increases in bone formation in cancellous and endocortical bone, accompanied by a sustained reduction in bone resorption. These effects lead to significant improvements in trabecular bone volume, microarchitecture, and cortical thickness after 12 months of treatment (35). Although animal studies suggest increased modeling bone formation in response to sclerostin inhibition, the relative contributions of remodeling and modeling to bone formation in humans remain to be fully elucidated (34).

### 2.3 Adverse effects of osteoporosis pharmacotherapies

Therapeutic interventions for osteoporosis, while effective in fracture prevention, are associated with distinct adverse effect profiles that necessitate careful risk-benefit evaluation. Bisphosphonates, including alendronate and zoledronate, are linked to gastrointestinal complications such as esophagitis and gastric ulceration, particularly with oral formulations, due to delayed mucosal healing exacerbated by gastroesophageal reflux (36, 37). Intravenous zoledronate frequently induces transient acute-phase reactions (30% incidence), characterized by myalgia, fever, and cytokine-driven inflammation, which typically resolve within days (38, 39). Although early studies suggested a potential association with atrial fibrillation, subsequent meta-analyses found no statistically significant risk (40–42). Ocular adverse events, including uveitis (0.8–1.1%), occur rarely but warrant patient counseling, as symptoms often emerge within days post-infusion and respond to topical therapies (43–45). Prolonged bisphosphonate use elevates the risk of atypical femoral fractures (AFFs), with adjusted relative risks of 1.70 (95% CI 1.22–2.37) in cohort studies, disproportionately affecting Asian populations and escalating with treatment duration beyond 3–5 years (46–48). Osteonecrosis of the jaw (ONJ), though rare in osteoporosis (0.01–0.001%), rises significantly in oncology settings (1–15%) due to high-dose regimens, comorbid therapies (e.g., glucocorticoids, antiangiogenics), and invasive dental procedures (49–52). Pathophysiological mechanisms involve suppressed bone remodeling, osteocyte apoptosis, and immune modulation via γδ T-cell dysregulation (53–56).

Denosumab, a RANKL inhibitor, demonstrates comparable antifracture efficacy but carries risks of hypocalcemia, necessitating pre-treatment vitamin D optimization (57). While AFFs (1:10,000) and ONJ remain uncommon in osteoporosis, extended use (7–10 years) correlates with incremental ONJ incidence, albeit far lower than in malignancy cohorts (57). Teriparatide, the sole anabolic agent, exhibits favorable tolerability with transient nausea and dizziness reported; rodent osteosarcoma findings have not translated to human risk (58, 59). Selective estrogen receptor modulators (SERMs), notably raloxifene, reduce vertebral fractures but increase thromboembolic events and fatal stroke risk, offsetting benefits in non-osteoporotic populations (60, 61).

Mitigation strategies emphasize pre-therapeutic dental evaluations to minimize ONJ risk, vigilance for prodromal AFF symptoms (e.g., thigh pain), and individualized duration limits for antiresorptives (62, 63). Clinicians must weigh skeletal benefits against context-specific harms, particularly in patients with comorbidities or extended treatment histories.

### 2.4 Pathogenesis of osteoporosis

#### 2.4.1 Gut microbiota in osteoporosis

The gut microbiota (GM), a diverse consortium of over 1,000 microbial species, emerges as a pivotal regulator of skeletal homeostasis. Comparative studies in germ-free (GF) mice reveal elevated trabecular bone mineral density (BMD), underscoring the GM’s role in physiological bone remodeling (64). Mechanistically, GM depletion attenuates osteoclastogenesis via reduced T-cell proliferation and proinflammatory cytokines (TNF-α, IL-6), while butyrate—a microbial-derived short-chain fatty acids (SCFAs)—enhances bone formation by stimulating osteocalcin secretion and Wnt10b signaling through Treg-CD8 + T cell crosstalk (65, 66). Intriguingly, butyrate supplementation rescues the anabolic effects of intermittent parathyroid hormone (iPTH) in microbiome-depleted models, restoring trabecular bone volume (67). Conversely, continuous PTH (cPTH)-induced bone loss requires GM-mediated expansion of pro-osteoclastic TNF + T and Th17 cells, highlighting the microbiota’s dual role in bone dynamics (68). These findings position GM modulation as a therapeutic frontier for osteoporosis (OP).

#### 2.4.2 Autophagy dysregulation: a nexus of bone cell dysfunction in osteoporosis

Autophagy, a conserved cellular recycling process, critically balances bone formation and resorption. In osteocytes, fluid shear stress enhances autophagic flux, preserving cellular viability under mechanical strain (69). Conversely, RANKL-induced osteoclast differentiation necessitates autophagy activation; its pharmacological inhibition (e.g., via chloroquine) mitigates glucocorticoid-driven bone loss by modulating RANKL/OPG ratios (70). Aging exacerbates OP pathogenesis through declined autophagy in bone marrow mesenchymal stem cells (BMMSCs), skewing differentiation toward adipogenesis via ROS/p53 pathways (71). Mitophagy defects further impair BMMSC function, linking mitochondrial dysfunction to senescent phenotypes (72). Thus, autophagy modulation represents a dual-edged yet promising target for OP intervention.

#### 2.4.3 Cellular senescence and the aging skeleton

Cellular senescence, marked by irreversible cell-cycle arrest and senescence-associated secretory phenotype (SASP) production, accumulates in aged bone microenvironments. Senescent osteoblasts, osteocytes, and immune cells exhibit elevated p16Ink4a expression, correlating with trabecular deterioration (73). Senolytic strategies—genetic ablation or pharmacological agents like ruxolitinib—reduce senescent burden, improving bone microstructure and strength in aged models (74). GM dysbiosis in senescence-accelerated mice parallels β-galactosidase upregulation and IL-17A-mediated BMMSC dysfunction, implicating microbial-metabolic crosstalk in age-related OP (71). Iron overload exacerbates senescence via ROS overproduction, reversible by chelators like Desferal^®^, which restore BMMSC osteogenic capacity (75). Targeting senescence thus offers a viable route to counteract OP progression.

#### 2.4.4 Therapeutic horizons: bridging mechanisms to clinical translation

Emerging therapies exploit GM modulation, autophagy enhancement, and senolysis to restore bone homeostasis. Probiotics and SCFAs supplementation may rectify GM dysbiosis, while autophagy inducers (e.g., rapamycin analogs) could rejuvenate BMMSC function (64). Senolytics, though nascent, show pre-clinical efficacy in eliminating senescent cells and mitigating SASP-driven bone loss (76). Animal models, particularly ovariectomy (OVX) and glucocorticoid-induced OP, remain indispensable despite limitations in replicating human pathophysiology (64, 77). Standardizing these models and integrating multi-omics approaches will accelerate translational breakthroughs.

## 3 Probiotics

Probiotics, when administered at physiologically effective concentrations, confer health benefits to the host organism, positioning them as a key focus of public health research (78). The expanding comprehension of dynamic crosstalk between enteric microbiota and host immune homeostasis has propelled investigations into probiotics’ immune-homeostatic regulatory functions, now constituting a pivotal research frontier in immunometabolism (79). Over the past several decades, the impact of probiotics on human health has been extensively studied by researchers, as well as the food and drug industries.

The concept of probiotics was initially associated primarily with beneficial bacteria. However, recent advancements have expanded the definition of probiotics to include not only bacteria but also other microorganisms, such as yeasts (80). Probiotics, including combined *Bifidobacterium*, *Saccharomyces boulardii* sachets, and *Bifid.Triple* Viable Capsules, are widely used in the treatment of diarrhea and various other diseases, particularly in children. A notable example is *Saccharomyces boulardii*, a yeast extensively studied for its positive effects on gastrointestinal health (81). Industrial-scale probiotic production pre-dominantly utilizes lactic acid bacteria (LAB) genera (*Leuconostoc, Lactobacillus, Lactococcus, Streptococcus, and Enterococcus*), spore-forming *Bacillus* species*, Bifidobacterium* spp.*, Propionibacterium* strains, and select non-pathogenic *Escherichia coli (E. coli)* variants (82). The enteroprotective mechanisms of probiotics operate through enhancing mucosal barrier integrity via stimulation of enterocyte mitogenesis, and maintaining selective paracellular transport through stabilization of intercellular junction complexes (83). Furthermore, Probiotics further strengthen the gut’s defenses by stimulating the production and secretion of antimicrobial peptides (84).

*Lactic acid bacteria* (LAB), a ubiquitous cluster of Gram-positive, non-pathogenic 2microorganisms, are prevalent in the human gut and fermented foods, and they are generally regarded as safe. Lactobacilli, a prominent subgroup of LAB, are frequently used in functional foods to regulate glucose and lipid metabolism, maintain gut microbial balance, and enhance host immune function (85, 86). Well-known strains include *Lactobacillus acidophilus, Lactobacillus casei, Lactobacillus plantarum, Lactobacillus rhamnosus, Lactococcus lactis, Bifidobacterium longum, Bifidobacterium infantis and Streptococcus thermophilus* (87). These bacteria have a long history in food fermentation, enhancing quality, flavor, texture, and promoting beneficial biological activities. For example, fermentation with *Lactobacillus plantarum WLPL01* increases the organic acid content and reduces the bitterness of Artemisia (88). Additionally, fermentation with *Bifidobacterium infantis* significantly altered the volatile and non-volatile components of barley juice and improved its antioxidant capacity (89). The advent of metabolomics technology has markedly heightened interest in the study of LAB-secreted bioactive compounds, including SCFAs, bacteriocins, and extracellular polysaccharides (EPS), due to their acknowledged significance in host physiology. The critical role of LAB in mammalian biology has been confirmed by studies conducted on sterile mice and antibiotic-treated mouse models (90, 91). Mechanistically, LAB execute their probiotic functions through three fundamental pathways: Alleviation of oxidative stress, preservation of intestinal epithelial barrier architecture, and homeostatic regulation of gut microbial ecology (86).

### 3.1 Genetic engineering paradigms in lactic acid bacteria

Plasmid-based systems remain foundational for LAB engineering yet face intrinsic constraints including low transformation efficiency due to Gram-positive cell wall barriers, strain-specific restriction-modification systems, and plasmid instability (92). Shuttle vectors like pTRKH2 and pLEM415-ldhL-mRFP1 circumvent these limitations through *E. coli*-LAB compatibility, enabling high-copy replication and functional gene expression tracking *in vivo* (93, 94). However, antibiotic selection pressures risk genomic mutation and microbiome dysbiosis, motivating chromosomal integration strategies. Early approaches utilized Rec-independent insertion (pTRK327) or homology-directed systems (pTRK685/pGK12) for stable gene insertion (95, 96).

The Cre-*lox* system advanced precision editing by enabling site-specific recombination, though its utility is constrained by off-target effects and screening complexity (97). CRISPR-Cas platforms now dominate LAB engineering through multiplexed editing capabilities. Pioneering work in *Lactobacillus reuteri* demonstrated Cas9-mediated double-strand break repair with homology-directed templates, while innovations like the all-in-one pNZDual plasmid reduced metabolic burden in *Lactococcus lactis* (98, 99). CRISPRi further enables tunable gene regulation, synergizing with the well-characterized NICE system for nisin-inducible protein secretion (100, 101). These tools collectively facilitate proteome optimization and metabolic pathway engineering while mitigating escape mutations through lethal selection (102, 103).

### 3.2 Precision probiotics

The burgeoning field of probiotics has transitioned from generalized formulations toward precision therapeutics, driven by the recognition of individual variability in gut microbiota composition, host physiology, and microbial interaction networks (13, 42, 43). Conventional probiotics, while beneficial for broad-spectrum dysbiosis, often fail to address patient-specific pathologies due to heterogeneous factors such as age, dietary habits, and immune responses. Precision probiotics address this gap by leveraging strain-specific molecular mechanisms—effector proteins, metabolites, and host-microbe signaling pathways—to restore targeted dysbiotic states.

Notable examples illustrate this mechanistic specificity: Precision strategies exploit molecular effectors such as *Lactobacillus helveticus* and *Bifidobacterium longum*-derived Runx2/BMP-2 upregulation to enhance osteogenesis (104, 105), and*Lactobacillus plantarum* mediated elevation of vitamin D receptor coupled with cytoprotective autophagy induction, synergistically promoting osteoblast activity (106, 107) while *Faecalibacterium prausnitzii* reinforces intestinal barrier integrity via butyrate production, mitigating inflammatory conditions (48). Similarly, *Bacillus amyloliquefaciens* demonstrates anti-osteoporosis effects mediated by the increased IGF-1 levels (108). These findings underscore the necessity of multi-omics integration (genomics, proteomics, metabolomics) and machine learning algorithms to decode strain-host synergies, enabling predictive selection of probiotics aligned with individual microbial ecosystems.

Advancements in computational biology facilitate patient stratification based on microbial biomarkers and clinical phenotypes, allowing tailored therapeutic regimens. AI-driven models analyze genomic signatures, metabolite profiles, and host metadata to predict colonization efficacy and monitor disease progression dynamically (45, 49). However, challenges persist in standardizing omics data across diverse cohorts and establishing universal functionality biomarkers. Collaborative efforts bridging clinical microbiology, bioinformatics, and systems biology are critical to realizing precision probiotics’ translational potential.

### 3.3 AI/ML-Driven advancements in probiotic research

Artificial intelligence (AI) and machine learning (ML) are revolutionizing probiotic development by enhancing precision across *in vitro*, *in silico*, *in vivo*, and clinical research paradigms. These technologies accelerate strain identification, functional characterization, and therapeutic optimization while minimizing human error and resource expenditure (109, 110).

#### 3.3.1 Strain screening and functional decoding

AI models like ABIOME simulate gut ecosystems using adaptive regression algorithms (MARS), identifying synergistic probiotic combinations while detecting antagonistic interactions (e.g., competitive amino acid depletion between *L. reuteri* and *Saccharomyces boulardii*) (111, 112). Concurrently, ML predicts excipient compatibility, optimizing formulations—such as 111 pharmaceutical agents enhancing *L. paracasei* viability (113).

ML algorithms enable high-throughput discrimination of probiotic candidates from non-probiotic microbes. Platforms like iProbiotics employ SVM classifiers and k-mer feature selection to achieve species-specific probiotic identification (110), while ANN models demonstrate 90% accuracy in tRNA sequence-based classification (112). Beyond taxonomy, ML integrates multi-omics data—such as transcriptomic networks of *Lactobacillus reuteri*—to elucidate strain-specific mechanisms (e.g., antimicrobial gene clusters) (114). Such *in silico* predictions synergize with *in vitro* assays; for instance, supervised ML screening of 144 LAB strains identified four *Lactobacillus* isolates with potent antimicrobial activity (115).

#### 3.3.2 Pre-clinical and clinical integration

In preclinical models, AI enhances pathological analysis efficiency, exemplified by deep learning-driven histopathological interpretation in murine colitis (116) and tumor phenotyping in lung adenocarcinoma (114). Clinically, AI/ML holds promise for stratifying patient populations and predicting therapeutic outcomes. A landmark study applied ML to 70,000 IBD patients, achieving robust prediction of disease progression and risk scores (117). While randomized trials validate probiotic efficacy—*Bacillus subtilis* against *S. aureus* colonization (118), microbiota modulation in T2D (119)—AI-augmented clinical frameworks remain underexplored.

Bridging AI with multi-omics and clinical metadata will refine predictive models for personalized probiotic therapies. Prioritizing AI-driven trial designs could optimize dosing regimens, monitor real-time microbiota shifts, and forecast host responses, ultimately accelerating translational outcomes.

#### 3.3.3 AI/ML-Driven probiotics for osteoporosis: decoding molecular mechanisms to clinical translation

The anti-osteoporotic properties of probiotics stem from molecular interactions mediated by surface proteins and metabolites. Clinically robust lactic acid bacteria (LAB) exhibit bile tolerance (via transporter activity) (120) and high adaptability to gastrointestinal stressors (acid, osmolarity) (121), while their surface proteins govern mucosal adhesion (immunomodulation) (78) and pathogen exclusion (122). A paradigm is L. rhamnosus GG, whose P40/P75 proteins enhance intestinal barrier integrity by activating epidermal growth factor receptors (EGFR) (123). These proteins synergize with bacteriocins (lactacin B, bifidocin) and SCFAs to suppress pathogens (S. aureus, H. pylori), modulate gut ecology, and ultimately ameliorate osteoporosis through microbiota-bone axis regulation (124, 125).

Despite omics technologies (genomics, proteomics, metabolomics) mapping these interactions, clinical translation remains hindered by data complexity (126). AI/ML bridges this gap by decoding multidimensional patterns: DeepFilter resolves uncharacterized bacterial proteomes via spectral analysis (127); ML models predict diet-driven shiftsin Lactobacillus and Ruminococcus populations (128); and CNN-based TaxoNN correlates microbiome profiles with osteoporosis risk (129).

Although direct studies on AI-guided probiotic screening for osteoporosis remain in their nascent stages, and no direct literature currently exists on AI/ML-based strain selection for osteoporosis treatment, interdisciplinary approaches suggest novel pathways with translational potential. By transforming data into predictive biomarkers, AI/ML holds promise for enabling precision selection of probiotics with optimized stability, therapeutic efficacy, and bone-protective potential in the future, paving the way for tailored microbiome interventions in osteoporosis management.

### 3.4 Challenges

Despite their therapeutic promise, probiotics face clinical implementation barriers due to host non-responsiveness (36% efficacy in diarrhea trials) and heterogeneous outcomes driven by individual variations in diet, genetics, and microbiota composition (130). Personalized approaches require integration of multi-omics data (genomics, proteomics, metabolomics) and clinical metadata to identify microbial biomarkers and replenish underrepresented taxa—a process hindered by invasive sampling methods and incomplete gut microbiome profiling (131, 132).

## 4 Probiotic-driven mechanisms in osteoporosis modulation

### 4.1 Association between gut microbiota and bone formation

The human skeleton undergoes continuous remodeling through coupled actions of osteoclasts, which resorb aged bone, and osteoblasts, which deposit new bone. Tight regulation of this balance is critical to prevent pathological changes in bone mass or quality (133). Emerging evidence implicates the gut microbiome as a novel modulator of skeletal homeostasis, potentially through microbial metabolites that influence systemic pathways (134, 135).

It is through the modulation of tryptophan metabolism that gut bacteria play a crucial role in skeletal homeostasis. Bacterial indoleamine-2,3-dioxygenase-1 (IDO-1) initiates tryptophan catabolism, producing kynurenine (an aryl hydrocarbon receptor (Ahr) ligand that promotes regulatory T-cell (Treg) differentiation and interleukin-22 (IL-22) secretion by group 3 innate lymphoid cells (ILC3s), thereby modulating inflammatory responses) (136). Mice fed a tryptophan-deficient diet exhibited altered gut microbiota composition and compromised intestinal immunity, underscoring the importance of tryptophan-derived metabolites in immune-bone crosstalk (136). While these findings highlight microbial metabolic influence, the precise origins and identities of Ahr ligands remain unresolved.

Insulin-like growth factor 1 (IGF-1) represents another critical mediator of the gut-bone axis. Germ-free (GF) mice colonized with specific pathogen-free (SPF) microbiota showed elevated serum IGF-1 levels—produced pre-dominantly in liver and adipose tissue—accompanied by increased bone formation and resorption markers (137). Conversely, antibiotic-induced microbiota depletion reduced IGF-1 production and suppressed osteogenesis, reinforcing microbiota-dependent regulation of bone metabolism (137).

Hydrogen sulfide (H2S), generated by both host gastrointestinal cells and gut microbes, further contributes to skeletal homeostasis. GF mice demonstrated diminished serum and intestinal H2S levels, suggesting microbial origin of systemic H2S (137). Dysregulated H2S impairs calcium flux via altered sulfhydration of TRP calcium channels, disrupting kinase signaling essential for osteoblast/osteoclast differentiation from bone marrow mesenchymal stem cells (138, 139).

Clinical evidence supports these mechanistic insights. Mendelian randomization studies link Clostridiales and Lachnospiraceae taxa to bone mass alterations (140). In a cohort of 181 participants, Clostridium Cluster XIVa (Firmicutes phylum) abundance correlated with osteopenia/osteoporosis prevalence (140). Similarly, postmenopausal women with osteoporosis/osteopenia exhibited reduced gut microbiota diversity and elevated Lachnospira pectinoschiza (Lachnospiraceae family) levels compared to controls (140). These findings collectively position gut microbiota composition as a potential biomarker and therapeutic target for bone disorders.

### 4.2 Probiotic-mediated attenuation of inflammatory and oxidative pathways in osteoporosis

The gastrointestinal tract, housing the body’s largest immune cell repertoire, plays a pivotal role in bone metabolism through cytokine-mediated regulation of bone remodeling (141, 142). Emerging evidence reveals that intestinal dysbiosis, particularly antibiotic-induced microbiota alterations, modulates systemic inflammation by reducing lipopolysaccharide (LPS) levels and associated inflammatory cascades (143). This gut-bone axis presents promising therapeutic targets, with probiotics demonstrating multifaceted regulatory capacities.

Mechanistically, probiotic-derived bacteriocins attenuate inflammatory responses through COX-2-mediated modulation of NLRP3 inflammasome and NF-κB signaling pathways (144). Such anti-inflammatory actions not only preserve tissue integrity but also suppress osteoclast differentiation, counteracting the RANKL/OPG imbalance characteristic of osteoporosis-associated dysbiosis (38). Notably, specific probiotic strains exhibit strain-specific benefits: *Lactobacillus rhamnosus* enhances mucosal immunity through IgA-mediated pathogen exclusion (145, 146), while EPS-producing *Bifidobacterium 35624* preferentially inhibits osteoclast precursor fusion through IL-17 regulation (147).

The aging process exacerbates bone loss through redox imbalance mechanisms. Postmenopausal estrogen decline impairs antioxidant defenses (reduced superoxide dismutase, folate, and GSH-Px activity), leading to ROS accumulation that disrupts bone homeostasis via MAPK, NF-κB, and Wnt/β-catenin pathway dysregulation (148–151) Crucially, gut microbiota composition modulates mitochondrial biogenesis through CREB-dependent glutathione synthesis, with *Firmicutes/Bacteroidetes* ratio alterations potentially exacerbating ROS-mediated bone resorption (152). Probiotic metabolites like urolithin A demonstrate therapeutic potential by simultaneously reducing osteoclast NLRP3 inflammasome activation (via caspase-1 and GSDMD suppression) and enhancing antioxidant capacity (153–155). After discussing the immunomodulatory role of probiotics, let’s now turn to their impact on the neuroendocrine system, which also has significant implications for bone health.

### 4.3 Neuroendocrine modulation

**FIGURE 1:**
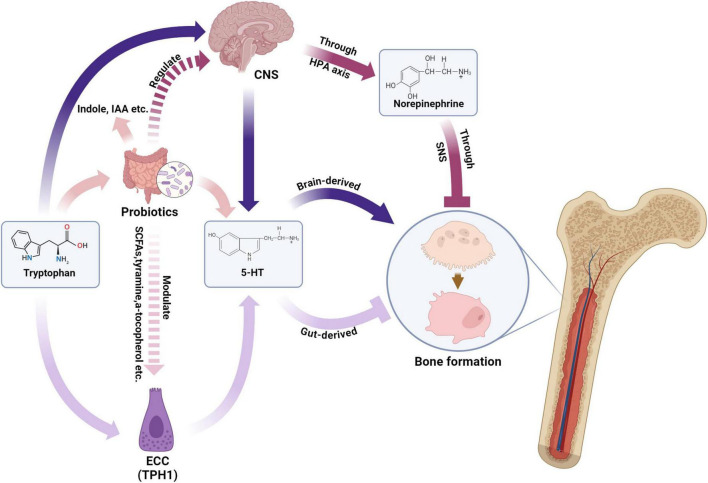
Probiotics modulate bone formation via gut-brain axis and neurotransmitters. NCC, enterochromaffin cell; TPH1, tryptophan hydroxylase 1; SCFA, short chain fatty acid; IAA, indoleacetic acid; 5-HT, 5-hydroxytryptamine; CNS, central nervous system; HPA axis, hypothalamic–pituitary–adrenal axis; SNS, sympathetic nervous system. Created in BioRender. Wenshuo, D. (2025) https://BioRender.com/nt3qm3r.

The bidirectional brain-gut axis integrates neural, endocrine, and immune pathways between the central nervous system and gastrointestinal tract (156, 157). Sensory neurons, gut hormones (e.g., 5-HT, GABA), and microbial metabolites mediate this crosstalk, with emerging evidence linking these interactions to bone homeostasis through osteocyte modulation (158–163). Probiotics demonstrate dual regulatory capacity: They restore gut microbiota equilibrium while influencing enteric nervous system activity, vagal signaling, and hypothalamic-pituitary-adrenal (HPA) axis dynamics—mechanisms potentially relevant to bone metabolism via neurotransmitter-mediated osteocyte regulation (164–167) (Figure 1).

#### 4.3.1 Serotonergic modulation

Gut-derived serotonin (g5-HT), synthesized from tryptophan in enterochromaffin cells, exerts site-specific effects on bone remodeling. Microbial metabolites critically modulate this pathway: Short-chain fatty acids upregulate tryptophan hydroxylase 1 (TPH1) to enhance g5-HT production, while specific gut bacteria (e.g., *Clostridium*, *Bacteroides*) metabolize tryptophan into indole derivatives that suppress g5-HT levels (168–174). This microbial-g5-HT crosstalk influences osteoblastogenesis through 5-HT1B receptor activation, with altered serotonin transporter (SERT) expression potentially disrupting bone-microbiome homeostasis.

#### 4.3.2 Adrenergic modulation

Norepinephrine (NE) from sympathetic neurons interacts with gut microbiota through cAMP-PKA-pCREB signaling, with elevated NE levels correlating with bone density suppression (175–177). Germ-free models reveal microbiota-dependent NE synthesis, as *Clostridium* colonization restores cecal NE concentrations (178). This microbial-neuroendocrine interplay suggests probiotics may modulate sympathetic tone through HPA axis regulation, though mechanistic details require further elucidation.

### 4.4 Endocrine modulation

Insulin-like growth factor 1 (IGF-1) serves as a central regulator of bone homeostasis through dual mechanisms: Stimulating osteoblast-mediated matrix synthesis and suppressing osteoclastic resorption (179). Emerging evidence highlights probiotics as potent modulators of IGF-1 bioactivity, offering therapeutic potential for osteoporosis. IGF-1 enhances bone anabolism by upregulating type I collagen synthesis, alkaline phosphatase activity, and osteocalcin expression while inhibiting collagen degradation (180, 181). Systemically, it coordinates mineral metabolism through renal phosphate reabsorption and calcitriol-mediated intestinal calcium/phosphate absorption (182).

Notably, specific gut microbiota strains exhibit strain-dependent capacity to activate the growth hormone/IGF-1 axis. *Lactobacillus plantarum* strains differentially enhance growth parameters in juvenile mammals, with LpWJL demonstrating superior efficacy in elevating hepatic IGF-1 expression and butyrate synthesis (183, 184). Mechanistic studies reveal that *Lactobacillus reuteri* attenuates diabetic bone loss via Wnt10b/IGF-1 crosstalk (185) while *Bacillus amyloliquefaciens* restores growth in malnourished models by amplifying nutrient absorption and mucosal immunity through GH/IGF-1 activation (108). SCFAs-producing taxa (e.g., Rikenellaceae, Clostridiales) further synergize this axis (186).

Of particular interest, *Bifidobacterium* species exhibit robust osteogenic effects. *B. longum* subsp. *infantis* CCFM1269 significantly elevates serum IGF-1 (*P* < 0.05) and osteogenic markers (OPG, osteocalcin) across sex and age groups, accompanied by increased IGFBP3 levels—a critical determinant of IGF-1 bioavailability (187).

### 4.5 Microbiota-derived metabolites modulation

#### 4.5.1 SCFAs and bone metabolism

SCFAs—primarily carboxylic acids with short hydrocarbon chains—are synthesized by the gut microbiota and can translocate from the gastrointestinal tract into systemic circulation. Once in circulation, SCFAs serve as pivotal signaling molecules in metabolism, immunity, and endocrine regulation (188–191, 192). Although early research mainly focused on the interactions of SCFAs with organs such as the liver, brain, pancreas, and kidneys, emerging evidence now suggests that SCFAs also play an important role in regulating bone metabolism (137, 191).

#### 4.5.2 Mechanisms by which SCFAs regulate bone metabolism

The mechanisms through which SCFAs regulate bone metabolism encompass the following: (1) Facilitating intestinal calcium absorption: SCFAs serve as an energy source for intestinal epithelial cells, maintaining barrier integrity. They enhance intestinal villus structure, increase epithelial surface area, and promote calcium absorption by improving the paracellular pathway and reducing pH in the intestinal lumen, which increases mineral solubility and supports bone formation (188, 193). (2) Modulating IGF-1 regulation: Another fundamental mechanism (137). (3) Regulating phytoestrogens: Gut bacteria, such as Bifidobacterium and Lactobacillus, metabolize phytoestrogens into compounds that bind estrogen receptors, promoting osteoblast proliferation, differentiation, and inhibiting osteoclast activity, thereby increasing bone mineral density (187, 194–196). (4) Inhibition of histone deacetylase (HDAC) activity: Butyrate exhibits HDAC inhibitory effects, facilitating the development of Foxp3 + regulatory T cells (Tregs), and contributing to immune system homeostasis (197, 198). (5) Interaction with G protein-coupled receptors (GPCRs): SCFAs modulate bone metabolism by reducing intracellular cyclic adenosine monophosphate (cAMP) levels, activating immune responses, promoting Treg proliferation, suppressing intestinal inflammation, and inhibiting osteoclast differentiation (199). (6) Regulation of 5-HT synthesis and release: SCFAs and secondary bile acids (2BAs) regulate 5-HT synthesis and release from enterochromaffin cells (ECCs), which interact with osteocytes to suppress osteoblast proliferation primarily through the activation of 5-HT1B receptors on preosteoblasts (200). (7) Vitamin D production and parathyroid hormone (PTH) secretion: Promote vitamin D production while inhibiting parathyroid hormone (PTH) secretion (201).

#### 4.5.3 Phytoestrogen metabolism

Recent research has highlighted several nutrients with potential to alleviate osteoporosis, with phytoestrogens being particularly notable (202, 203). Phytoestrogens are polyphenolic compounds found in plants such as soybean and flaxseed. They resemble mammalian estrogens structurally and can exert estrogen-like effects in biological systems (204). These compounds are primarily categorized into isoflavones, ellagitannin, and lignans, which undergo metabolism by gut bacteria to form more biologically active compounds like equol, urolithin, and enterolipin (205).

These metabolites exhibit enhanced estrogenic/antiestrogenic and antioxidant activities in comparison to their precursors (206). Furthermore, they possess anti-inflammatory, anti-proliferative, and pro-apoptotic effects (207). Studies have demonstrated that intestinal bacteria such as *Bifidobacterium* and *Lactobacillus* can metabolize isoflavones into equol, which mimics estrogen activities (208, 209). Equol bind to estrogen receptors ERα and ERβ, stimulates the differentiation of mesenchymal stem cells into immature osteoblasts, impedes the differentiation and activation of osteoclasts, induces their apoptosis, and restores the equilibrium between bone formation and resorption (210–213). Ellagitannin can be converted by gut bacteria, like *Clostridium leptum* and *Ruminococcus bromii*, into urolithin, a compound with antioxidant properties that may reduce inflammation, promote bone formation, and inhibit bone resorption (214–216). Additionally, *Clostridium*, *Klebsiella*, and *Collinia* participate in the conversion of lignans into enterolipins (especially enterodiol and enterolactone), which regulate hormone levels, particularly estrogen, and promote bone health (196, 217). However, it is important to note that the complex structure of the gut microbiota is critical for proper lignan metabolism. For instance, *Eggerthella lenta* does not independently convert secoisolariciresino (SECO) to enterolactone but does so in co-culture with *Blautia producta* (218, 219).

### 4.6 Gene expression modulation

**FIGURE 2:**
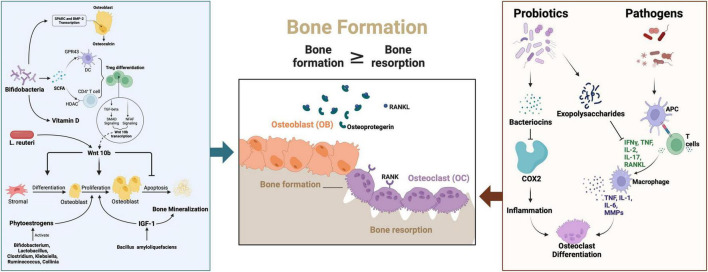
The signaling pathway of the probiotics and pathogens in the pathogenesis and probiotics therapies for osteoporosis. SPSRC, Secreted protein acidic and rich in cysteine; BMP-2, Bone morphogenetic protein 2; SCFA, short-chain fatty acids; RANKL, Receptor Activator of nuclear factor-kappa B ligand;APC, Antigen presenting cells; IFN-γ, Interferon-γ; IL-1, Interleukin -1; IL-2, Interleukin-2; IL-6, Interleukin-6;IL-17, Interleukin-17; TNF, Tumor Necrosis Factor; MMPs, Matrix metalloproteinases; COX-2, Cyclooxygenase-2; Pi, Phosphorus; NFAT, Nuclear factor of activated T cells; L. reuteri, Limosilactobacillus reuteri; IGF-1, Insulin-like growth factor 1. Created in BioRender. Wei, A. (2025) https://BioRender.com/e75e579.

Genes associated with bone calcification and remodeling - including SPARC (secreted protein acidic and cysteine-rich), the osteogenic master transcription factor RUNX2, and bone morphogenetic protein 2 (BMP-2) - are critical regulators of skeletal development (Figure 2). Emerging evidence indicates that probiotic interventions can modulate their expression to influence osteoporosis progression (105, 220). Specifically, SPARC functions as a calcium-binding matrix protein that facilitates bone mineralization through transcriptional activation of calcification-related targets during tissue repair and remodeling processes (221, 222).

Clinical investigations by Parvaneh et al. revealed that Bifidobacterium longum supplementation upregulated SPARC and BMP-2 expression, corresponding with increased serum osteocalcin (a bone formation marker) and decreased C-terminal telopeptide of type I collagen (CTX, a bone resorption indicator) (105). The Wnt signaling pathway, particularly through its key ligand Wnt10b, further contributes to osteoblast regulation and bone homeostasis (223). Mechanistically, probiotics enhance osteogenic Wnt10b production via butyrate-mediated differentiation of regulatory T cells (Tregs) (224). Supporting this pathway, Zhang et al. demonstrated that Lactobacillus reuteri administration prevented Wnt10b signaling suppression and maintained osteoblast activity in type 1 diabetic mice, establishing a direct association between Wnt10b dysregulation and diabetic osteoporosis progression.

## 5 Conventional probiotics

Emerging pre-clinical studies delineate a paradigm shift in osteoporosis management through targeted microbial modulation. Conventional probiotics (*Lactobacillus*, *Bifidobacterium*) demonstrate conserved osteoprotective mechanisms across animal models, including suppression of osteoclastogenesis via TNF-α/IL-1β inhibition and enhancement of intestinal barrier integrity through ZO-1/occludin upregulation (225–227). These findings align with *Saccharomyces* species’ capacity to attenuate bone resorption through RANKL/IL-17 axis modulation (228), and *Bacillus* strains’ dual action in T-cell polarization rebalancing and vitamin D metabolism potentiation (229, 230).

### 5.1 Lactobacillus

Although representing a minor proportion of gut microbiota (< 10% in duodenum, < 1% in colon) (231, 232), *Lactobacillus* species emerge as critical modulators of bone homeostasis through their unique acid tolerance and S-layer protein-mediated intestinal colonization (233, 234). Clinical evidence underscores their therapeutic potential: A landmark randomized trial (*n* = 249) demonstrated that tri-strain *Lactobacillus* supplementation preserved lumbar bone mass in postmenopausal women via WNT pathway modulation (WNT16, sFRP4, Wnt10b) (235), establishing a paradigm for microbiota-targeted interventions.

#### 5.1.1 Mechanistic interplay between lactobacilli and skeletal system

Lactobacilli orchestrate bone remodeling through three synergistic axes. First, their capacity to produce short-chain fatty acids enhances mineral bioavailability, exemplified by *Lactobacillus plantarum*-mediated 10% elevation in Caco-2 calcium absorption through vitamin D receptor upregulation and transcellular transport potentiation (107, 236). Second, lactobacilli reinforce intestinal barrier integrity via species-specific mechanisms: *L. plantarum* induces dose-dependent tight junction reorganization in colonic epithelia (237–239), while *L. rhamnosus* ameliorates mucosal damage through Th17/Treg balance restoration and inflammatory cytokine suppression (240). Third, direct osteogenic modulation occurs via strain-dependent signaling—*L. helveticus* upregulates Runx2/BMP-2 expression and serum osteocalcin levels (105), whereas *L. plantarum* stimulates pyrazine synthesis to activate osteogenic genes (OSX, osteocalcin) (106, 241).

#### 5.1.2 Clinical heterogeneity and therapeutic implications

Divergent clinical outcomes highlight the complexity of lactobacilli-bone interactions (Table 1). While *L. reuteri* NCIMB 30242 significantly elevated serum 25(OH)D in adults (242), its effects on bone density remain context-dependent. Notably, a 12-month RCT of *L. reuteri* 6,475 in osteopenic elderly women demonstrated improved trabecular bone mineral density (Tb.BMD: + 2.1% vs. −1.8% in placebo, *P* = 0.03) through gut microbiota modulation (a *Faecalibacterium*, ↓ *Escherichia*; *q* < 0.05) (11), yet a subsequent multi-omics analysis by Li et al. on the same cohort revealed non-responders exhibited detrimental microbial shifts, including enrichment of *Escherichia coli* (log2FC = 3.7, adjusted *P* = 0.04) and upregulated biofilm formation (e.g., *arcA*, *csgA*; *P* < 0.01), suggesting non-canonical pathways (243). Intriguingly, *L. acidophilus*-natto combinations paradoxically increased femoral calcium despite reducing serum levels, emphasizing tissue-specific bioavailability (236). These discrepancies underscore the necessity to delineate strain-specific pharmacodynamics and host-microbe crosstalk.

**TABLE 1:** Effect of probiotic supplementation on clinical trials.

Participants number	Intervention	Duration	Measurement	Major finding	Study
127 Healthy high-cholesterol adults	Administration group(G): G1: Control (13-week placebo) G2: L.reuteri NCIMB 30242(4-week placebo + 9-week L.reuteri NCIMB 30242)	13 weeks	1. Serum low-density lipoprotein- cholesterol 2. fat-soluble vitamins	L.reuteri NCIMB 30242 increased serum 25-OH vitamin D levels,which is precursor of active vitamin D. Active vitamin D can directly influence bone metabolism.	Jones et al. Canada (215)
70 Women who were 75−80 years old and had low BMD	G1: Control (placebo) G2: 1010 colony-forming units of L. reuteri 6475	12 months	1.Tibia total volumetric BMD	L. reuteri 6475 was effective in reducing total vBMD loss com-pared to placebo.	Nilsson et al. Sweden (11)
54 Postmenopausal women	G1: Control(soymilk) G2: soy-honey fermented milk [with *Lacto- bacillus casei* subsp. casei R-68 (SMH Lc)] G3: soy-honey fermented milk [with Lacto- bacillus plantarum 1 R 1.3.2 (SMH Lp)]	90 DAYS	1. Osteocalcin 2. Serum osteocalcin levels 3. Others (random blood glucose, uric acid, and total cholesterol levels)	Osteocalcin levels and cholesterol levels were significantly reduced by fermented soy honey containing SMH Lp and SMH Lc.	Desfita et al. Indonesia (216)
53 Participants who were older than 55 years	G1: Control [(microcrystalline cellulose) G2: *Probiotics capsule* L. fermentum SRK414, 4.0 × 10^9^ CFU]	6 months	1. BTMs 2. BMD 3. 0C	1. L. fermentum SRK414 improved Femur neck BMD in G2. 2. L. fermentum SRK414 maitained the level of OC in G2. 3. There was a significant correlation between changing L. fermentum concentrations and changing OC levels.	Han et al. Korea (217)
40 Postmenopausal women	G1: Control (placebo material, calcium, calcitriol) G2: Probiotic [Bifdobacterium animalis subsp. lactis Probio-M8 (Probio-M8), calcium, calcitriol]	3 months	1. Bone mineral density 2. Blood sample analysis (PTH, VD3, Ca^2+^, phosphorus, ALP, OC, tP1NP, B-CTX, PCT) 3. Fecal sample analysis	Co-administration of calcium, osteotriol and probiotic M8 has been shown to increase vitamin D3 levels and decrease serum levels of PTH and calcitoninogen	Zhao et al. China (218)
55 Participants who were 45−70 years	G1: Control (placebo) G2: probiotic (L. acidophilus UALa-01TM)	12 Weeks	1. Body composition 2. Blood biochemical parameters 3. Serum calcium levels 4. Biomarkers of bone metabolism	1. Serum calcium levels decreased in the probiotic group compared to baseline. 2. Taking L. acidophilus probiotics appears to help slow fluctuations in bone turnover markers.	Harahap et al. Poland (219)

BMD, bone mineral densitometry; BTM, bone turnover markers; OC, osteocalcin.

### 5.2 Bifidobacterium

As keystone commensals within gut microbiota, *Bifidobacterium* species (*B. longum*, *B. adolescentis*, *B. bifidum*) demonstrate emerging potential in osteoporosis intervention through multimodal biological pathways (244). Clinical evidence reveals that *B. longum* supplementation enhances vitamin D bioavailability and calcium absorption, with a phase III trial demonstrating significant elevation of serum vitamin D3 and reduction of procalcitonin in postmenopausal women receiving *Bifidobacterium*-calcitriol co-therapy *versus* controls (229, 245, 246). These effects are mechanistically linked to TLR2-dependent osteoclast inhibition mediated by *B. bifidum*-derived exopolysaccharides (247), alongside immunomodulatory capacity to suppress proinflammatory cytokine networks (248).

#### 5.2.1 Prebiotic synergy and ecological modulation

The therapeutic landscape extends beyond direct probiotic administration to encompass prebiotic strategies that selectively enrich endogenous *Bifidobacterium* populations. Phytochemical interventions—notably grape seed anthocyanins and konjac oligosaccharides—exert osteoprotective effects by reshaping microbial ecology. These compounds enhance *Bifidobacterium* abundance while suppressing opportunistic pathogens, concurrently restoring intestinal barrier integrity and bone marrow immune homeostasis (230, 249). Such findings position plant-derived prebiotics as ecological modulators that amplify the osteoanabolic potential of commensal bifidobacteria.

### 5.3 Saccharomyces

Emerging evidence positions *Saccharomyces* species—notably *S. boulardii* and *S. cerevisiae*—as novel modulators of osteoimmunological balance through pleiotropic mechanisms. The *S. boulardii* CNCM I-745 strain demonstrates potent anti-osteoclastic activity by suppressing RANKL/IL-17/TNFα signaling cascades, effectively attenuating inflammatory bone resorption (228). This immunometabolic modulation extends to direct interference with osteoclast differentiation through bone marrow monocyte interactions, establishing a dual therapeutic axis against pathological bone loss.

#### 5.3.1 Bioactive components and translational potential

Beyond classical probiotic functions, *Saccharomyces*-derived compounds exhibit targeted osteoprotection. β-Glucans from *S. cerevisiae* demonstrate systemic bone metabolism regulation, with pre-clinical studies highlighting their capacity to mitigate alveolar bone deterioration in metabolic disorder contexts (250). Notably, yeast hydrolyzates restore sex hormone equilibrium and trabecular microarchitecture, suggesting utility in postmenopausal osteoporosis management (251) A breakthrough innovation lies in yeast-conjugated gallium (YG), which synergizes enhanced bone mineral density with reduced osteoclast activity (evidenced by suppressed TRACP-5b), while circumventing elemental toxicity through organic complexation (252, 253).

### 5.4 Bacillus

As resilient Gram-positive probiotics, *Bacillus* species (*B. coagulans*, *B. subtilis*) emerge as potent modulators of osteoimmune homeostasis through multifaceted mechanisms (254, 255). Clinical evidence reveals that *B. coagulans* supplementation enhances bone mineral density, particularly in weight-bearing skeletal regions, via dual immunometabolic pathways: Suppression of bone-resorptive cytokines and potentiation of vitamin D biosynthesis (256). This vitamin D elevation—uncoupled from serum calcium fluctuations—suggests tissue-specific endocrine modulation rather than systemic mineral regulation.*B. subtilis* further expands this therapeutic repertoire by rebalancing T-cell polarization, specifically through Th17 suppression and Treg population expansion, thereby attenuating osteoclastogenic inflammatory cascades (IL-17, IL-6, TNF-α) (220, 256, 257).

While conventional probiotics have shown therapeutic promise, recent advancements in synthetic biology have enabled the engineering of next-generation probiotics with enhanced functionalities.

## 6 Next-generation probiotics

### 6.1 Revolutionizing probiotic therapeutics through synthetic biology

The integration of synthetic biology tools into probiotic engineering represents a paradigm shift in developing next generation biotherapeutics. By reprograming microbial chassis to dynamically sense pathological signals and execute therapeutic responses, researchers are advancing live biotherapeutic systems capable of treating metabolic disorders with unprecedented precision (19, 258). Notably, synthetic biology-engineered probiotics have demonstrated clinical potential in addressing homocystinuria, osseointegration, tumor microenvironment, and inflammatory bowel disease through targeted enzyme delivery and metabolite regulation (259–262).

These next-generation probiotics (NGPs) combine therapeutic enzyme expression with sophisticated biosensing circuits, enabling real-time detection and mitigation of disease biomarkers within the gastrointestinal microenvironment (263). However, their translational implementation faces critical biological barriers. The gut ecosystem imposes dual challenges through both physicochemical stressors and microbial competition, where commensal microbiota outcompetes therapeutic strains for nutritional resources while creating colonization resistance (264). Furthermore, maintaining microbial viability and functional stability within the intestinal lumen’s dynamic conditions remains a pivotal hurdle for sustained therapeutic efficacy.

### 6.2 Escherichia coli Nissle 1917 (EcN): a versatile platform for engineered biotherapeutics

First isolated during a World War I shigellosis outbreak, *E. coli* Nissle 1917 (EcN) demonstrated intrinsic resistance to enteric pathogens, later validated for its immunomodulatory, anti-inflammatory, and antimicrobial properties (265, 266). Marketed as Mutaflor^®^, EcN is clinically proven to alleviate acute diarrhea in pediatric populations (267) and ulcerative colitis (UC) symptoms comparably to mesalazine (268). Its efficacy stems from anti-inflammatory cytokine induction and competitive exclusion of pathogens via microcin H47 secretion (269, 270). These traits, combined with genomic stability and a long safety profile, position EcN as a robust chassis for NGPs (271).

#### 6.2.1 Genetic toolbox and biocontainment strategies

EcN’s fully annotated genome (272) and cryptic plasmids (pMUT1/2) enable stable heterologous expression without antibiotic selection (273, 274). Chromosomal integration further enhances genetic stability (275), while conjugation-based systems improve transformation efficiency (276). To address biocontainment concerns, CRISPR-Cas9 kill switches and temperature-sensitive circuits ensure controlled proliferation and environmental safety (277). These advancements support EcN’s application in drug delivery (278), biosensing (279), and inflammatory disease mitigation (280).

#### 6.2.2 Therapeutic applications of engineered EcN

EcN’s modularity enables tailored therapies for diverse domains. In oncology, engineered strains enhance antitumor immunity through L-arginine-mediated T-cell infiltration (281) or STING pathway activation via cyclic di-AMP delivery (282). Anti-infective strategies leverage pathogen-specific mechanisms, including tetrathionate-responsive microcins against Salmonella (122) and bile salt hydrolase-mediated inhibition of C. difficile sporulation (258). Emerging applications span neuromodulation (GABA production via gadB overexpression for neuropsychiatric disorders) (283) and ethanol detoxification through metabolic pathway engineering (284).These advances highlight EcN’s versatility as a therapeutic platform, with multiple candidates progressing toward FDA approval.

However, current clinical research remains largely confined to pre-clinical studies and early phase trials. Despite demonstrating promising experimental efficacy, substantial barriers persist before widespread clinical adoption can be realized.

Illustrating this translational challenge, Synlogic’s EcN-based engineered strain SYNB1934—developed for phenylketonuria—achieved a 34% reduction in plasma phenylalanine during its Phase II trial (NCT04534842) (285). Nevertheless, its pivotal Phase III study (NCT05764239) was terminated in 2024 due to suboptimal efficacy.

Ongoing efforts aim to address unmet needs in homocystinuria and enteric hyperoxaluria using the EcN platform. For SYNB8802 (targeting hyperoxaluria), pre-clinical work by Lubkowicz et al. successfully modeled and predicted clinically relevant urinary oxalate reductions (> 20%) (286). Yet, while its Phase I trial (NCT04629170) has completed, results remain unpublished. Similarly, the Phase I trial of SYNB1353 for homocystinuria (NCT05462132) demonstrated a statistically significant 26% reduction in plasma methionine (AUC_0–24_)(*p* < 0.05) in methionine-loaded healthy volunteers (260). This preliminary finding warrants validation in homocystinuria patients, and further Phase II studies are planned.

Despite EcN’s versatile therapeutic adaptability and the clinical advancement of multiple candidates, collective data reveal unresolved translational barriers that challenge the platform’s broader applicability.

### 6.3 Emerging non-conventional probiotics

Novel probiotic candidates such as *Akkermansia muciniphila*, *Faecalibacterium duncaniae*, *Bacteroides fragilis*, and *Bacillus clausii* are gaining attention for their therapeutic potential in inflammatory and metabolic disorders (287–290). These species contribute to gut homeostasis through distinct mechanisms, notably via the biosynthesis of SCFAs including acetate, propionate, and butyrate. *A. muciniphila* enhances metabolic health by stimulating glucagon-like peptide-1 (GLP-1) secretion, a mechanism linked to improved glycemic regulation in murine models (291). Genomic characterization of this species has further identified redundant mucinase genes, suggesting an evolutionary adaptation for mucin degradation and niche colonization (292)

*F. duncaniae*, a dominant butyrogenic commensal, exerts anti-inflammatory effects by IL-10-secreting, Foxp3-expressing T regulatory cells, thereby attenuating mucosal inflammation (288). Similarly, *Bacillus clausii* modulates glucose fermentation dynamics through propionate production, influencing host metabolic pathways (293). Within the *Bacteroides* genus, species such as *B. fragilis*, *B. thetaiotaomicron*, and *B. vulgatus* exhibit competitive fitness in the gut ecosystem via polysaccharide utilization loci (PULs), enabling efficient catabolism of complex dietary fibers (294, 295). However, their clinical application is complicated by strain-specific virulence factors; for instance, enterotoxigenic *B. fragilis* variants are implicated in colorectal carcinogenesis through bacteriocin and toxin production (296).

To harness their therapeutic potential, targeted gene editing—such as deletion of virulence determinants or heterologous expression of carbohydrate-active enzymes—may enhance safety and colonization efficacy. Nevertheless, developing organism-specific genetic toolkits remains a critical hurdle for engineering these phylogenetically diverse candidates.

### 6.4 Evolutionary advancements in microbial therapeutics

This mechanistic convergence underpins the therapeutic potential of next-generation probiotics like *Anaerostipes caccae*, a spore-forming Lachnospiraceae member that optimizes butyrate production and oxygen tolerance (297, 298). Comparative pre-clinical analyses reveal *A. caccae*’s superior biodurability and metabolic versatility. Long-term supplementation in avian models demonstrated sustained trabecular preservation through bone marrow immunomodulation, outperforming conventional probiotics in aging-related bone loss attenuation (299). Synergistic formulations with lactulose amplify butyrate synthesis, achieving dual osteoprotective and anti-allergic effects in gnotobiotic systems—a therapeutic breadth unmatched by first-generation probiotics (300).

### 6.5 Advancing genetic toolkits for engineering non-conventional probiotics

The therapeutic potential of non-conventional probiotics remains constrained by the scarcity of organism-specific genetic engineering platforms. While synthetic biology strategies established for model probiotics offer a foundational framework, their adaptation to phylogenetically diverse species requires systematic optimization. For instance, *Akkermansia muciniphila*—a mucinolytic specialist producing immunomodulatory SCFAs—has been engineered using a codon-optimized Himar1 transposase system (301, 302). This approach enabled the creation of a transposon mutagenesis library, revealing that mucin degradation machinery is critical for both glycan metabolism and gastrointestinal colonization (292).

CRISPR-based systems have demonstrated species-specific challenges and opportunities. In *Clostridium butyricum*, initial attempts to enhance butyrate yields via heterologous *Streptococcus pyogenes* Cas9 faced toxicity limitations (303). By contrast, leveraging the endogenous Type I-B CRISPR-Cas system improved editing efficiency, achieving a 60% increase in butyrate production through targeted knockout of *spo0A* and *aldh* genes (304). Similarly, *Bacteroides* spp.—dominant gut colonizers harboring both metabolic versatility and pathogenic potential—exhibit diverse native CRISPR systems (Type I-B, III-B, II-C) that could be repurposed to silence virulence factors or xenogeneic elements (305, 306).

Beyond genetic manipulation, understanding host-microbe interactions remains pivotal. Innovative platforms like the Gut Microbiome Physiome (GuMI) system have elucidated how butyrate-producing strains modulate inflammatory pathways, including TLR3/4 downregulation (307). Future efforts to expand the genetic toolbox for NGPs should prioritize both precision genome editing and functional validation within physiologically relevant models, ultimately enabling tailored expression of therapeutic biomolecules.

### 6.6 Reassessing probiotic safety and translational challenges

Despite their therapeutic promise, probiotic interventions carry non-trivial risks ranging from subclinical inefficacy to severe adverse events. A landmark Dutch trial administering multispecies probiotics to acute pancreatitis patients reported higher incidence of bowel ischemia and mortality compared to placebo—a cautionary outcome that significantly impacted clinical probiotic research trajectories (308, 309). Beyond acute complications, chronic issues such as D-lactic acidosis from LAB-derived metabolites can induce neurocognitive impairments, while post-antibiotic probiotic use may paradoxically delay microbiome reconstitution (310, 311). Safety concerns are compounded by risks of bacteremia, horizontal antibiotic resistance transfer, and unintended ecological disruption in antibiotic-treated hosts (312, 313).

These challenges underscore the need for precision engineering in NGPs. Advanced genetic tools could mitigate risks by eliminating virulence factors and enhancing strain stability (314). For instance, Novome Biotechnologies employs synthetic biology to engineer *Bacteroides* strains with tailored polysaccharide utilization loci, coupled with prebiotic porphyran supplementation to create selective metabolic niches (308, 315). Such synbiotic strategies may overcome colonization barriers that frequently undermine therapeutic efficacy in late-stage trials (316). By integrating strain optimization with ecological engineering, researchers can address both safety and functionality gaps in probiotic development.

## 7 Bacterial extracellular vesicles (BEVs): emerging therapeutic agents in OP management

### 7.1 Biogenesis and functional diversity of BEVs

BEVs, bilayered spherical nanostructures (20–400 nm diameter), encapsulate diverse cargo including glycoproteins, enzymes, nucleic acids, and metabolites, enabling their roles in immunomodulation, microbial colonization, and metabolic cooperation (317, 318). Initially identified in Gram-negative bacteria in the 1960s (319), BEVs were later recognized in Gram-positive species in the 1990s (320). Their biogenesis mechanisms differ markedly between bacterial classifications: Gram-negative bacteria produce outer membrane vesicles (OMVs) via membrane blebbing or explosive cell lysis (EOMV/OIMV), while Gram-positive counterparts generate cytoplasmic membrane vesicles (CMVs) through programed cell lysis pathways (23, 321, 322). Compositionally, OMVs are enriched with outer membrane proteins influenced by cell wall dynamics, whereas CMVs and EOMV/OIMV harbor peptidoglycan, nucleic acids, and cytoplasmic components due to their lytic origins (18, 217). A critical distinction lies in the exclusive presence of lipopolysaccharide (LPS) in Gram-negative BEVs, a feature linked to both therapeutic potential and systemic toxicity (23, 321).

### 7.2 BEVs as therapeutic platforms for OP

BEVs represent a promising experimental therapeutic strategy for systemic bone diseases such as OP, leveraging their cell-free nature, nanosized architecture, biocompatibility, and non-replicative properties. Compared to parental probiotics, BEVs may offer enhanced safety and efficacy potential in modulating the “gut-bone” axis—a critical pathway involving intestinal metabolites, immune regulation, and endocrine signaling (323, 324). Notably, BEVs derived from probiotics like *Akkermansia muciniphila*, *Lactobacillus* spp., and *Bifidobacterium* spp., which demonstrate pre-clinical anti-osteoporotic effects in models, represent potential candidates for OP treatment that require further validation.

OP fracture treatment demands a holistic approach addressing bone loss, microenvironment repair, and mechanical stabilization. Pre-clinical studies suggest that BEVs can synergize with mesoporous inorganic biomaterials (325, 326), metallic scaffolds (327, 328), and hydrogels (329) to enhance fracture repair. As dual-purpose nanocarriers in experimental settings, BEVs have shown potential to simultaneously deliver anti-resorptive agents and promote osteogenic differentiation, addressing both systemic OP and localized fracture healing in animal models..

### 7.3 BEVs in osteoporosis: mechanisms and engineering advances

BEVs derived from probiotic or attenuated bacterial strains show pre-clinical promise in bone disease therapeutics by circumventing LPS-mediated toxicity. For instance, *Proteus mirabilis* (PM)-derived BEVs were shown to suppress osteoclastogenesis by elevating reactive oxygen species (ROS), disrupting mitochondrial membrane potential, and modulating apoptosis-related proteins (Bax, Bcl-2, Caspase-3) (330). In ovariectomized (OVX) mice, PM-BEV administration mitigated bone loss, highlighting their experimental osteoprotective potential (330).

Engineering strategies further enhance BEV functionality. Liu et al. engineered BEVs expressing BMP-2 and CXCR4 fused to ClyA surface proteins, which promoted BMSC osteogenic differentiation in OVX models (331). To address poor bone targeting, bone-homing peptides were anchored to *Lactobacillus rhamnosus* GG-derived EVs (LGG-EVs), enabling miRNA delivery to bone microenvironments while inhibiting osteoclastogenesis (332). Similarly, anti-miR-6359-loaded exosomes modified with EXOmotif (CGGGAGC) demonstrated precise osteoclast precursor targeting (333). These pre-clinical innovations underscore the versatility of engineered BEVs in balancing efficacy and safety, but remain to be translated clinically.

### 7.4 Engineering strategies for functionalizing bacterial extracellular vesicles (BEVs)

#### 7.4.1 Physical engineering approaches

##### 7.4.1.1 Membrane fusion

Liposome-mediated fusion stands as a versatile method to enhance BEV functionality. Simple incubation at 37°C enables EV-liposome fusion (334), while polyethylene glycol (PEG) facilitates efficient hybridization (335). For example, fusion of CXCR4-engineered MEVs with antagomir-188-loaded liposomes yielded hybrid nanoparticles with dual bone-targeting and therapeutic capabilities (336). Similarly, Lin et al. demonstrated CRISPR-Cas9 delivery to mesenchymal stem cells (MSCs) via MEV-liposome hybrids (334), highlighting the potential for BEVs to integrate gene-editing tools.

##### 7.4.1.2 Membrane coating

Nanoparticle encapsulation through membrane coating enhances BEV functionality while preserving targeting specificity. Chen et al. engineered BEVs to coat indocyanine green (ICG)-loaded mesoporous silica nanoparticles (MSNs), achieving targeted dendritic cell delivery (337). Hybrid membrane systems, such as BEV-cancer EV (CEV) fusions, further combine tumor-targeting properties with immunogenicity for precision drug delivery (338). These platforms exemplify how BEV-coated nanoparticles could bridge therapeutic and diagnostic applications.

##### 7.4.1.3 Electroporation

Electroporation enables efficient cargo loading without compromising vesicle integrity. Optimized conditions (400 V, 125 μF in potassium phosphate buffer) successfully introduced siRNA into MEVs for neuron-specific delivery via Lamp2B-RVG targeting (339) Applied to BEVs, this method could load antiresorptive or anabolic agents for osteoporosis therapy. Notably, Zha et al. demonstrated VEGF plasmid delivery via progenitor cell-derived MEVs to enhance bone repair (340), suggesting electroporation’s adaptability for BEV-based regenerative strategies.

#### 7.4.2 Chemical engineering approaches

Chemical modification strategies for BEVs are broadly classified into covalent and non-covalent methods, each offering distinct advantages in precision, stability, and applicability. Covalent approaches leverage robust chemical bonds to permanently functionalize BEV surfaces, while non-covalent strategies prioritize flexibility and simplicity for transient interactions. Below, we dissect these methodologies and their implications for BEV engineering.

##### 7.4.2.1 Covalent modifications

Covalent engineering exploits stable chemical bonds to anchor functional moieties onto BEV membranes. *Click chemistry*, for instance, enables site-specific conjugation of targeting peptides or imaging probes through bioorthogonal reactions, minimizing off-target effects (341). Huang et al. demonstrated this principle by tethering quantum dots to MEVs using DNA hinges, achieving precise labeling without compromising vesicle integrity (342). Similarly, *aldehyde-amine condensation* and *amidation reactions* facilitate aptamer conjugation, transforming BEVs into smart platforms for precision therapeutics (343). Bioconjugation strategies further capitalize on BEV-specific surface markers, such as CD63, to anchor functional peptides like CP05, thereby enhancing cargo-loading efficiency (344). However, the limited identification of BEV-specific markers—compared to MEVs, which express well-characterized tetraspanins (e.g., CD9, CD81) and TSG101 (345)—remains a bottleneck. Additionally, the long-term biocompatibility and immunogenicity of covalently modified BEVs warrant rigorous investigation to ensure clinical viability.

##### 7.4.2.2 Non-covalent strategies

Non-covalent modifications offer reversible and rapid functionalization, ideal for applications requiring dynamic interactions. Hydrophobic insertion, a widely adopted method, exploits the amphiphilic nature of BEV membranes. For example, DSPE-PEG derivatives conjugated to targeting ligands (e.g., RGD, folate) spontaneously integrate into lipid bilayers, enhancing BEV homing to specific tissues (346). Electrostatic interactions provide another avenue: Cationic polymers or lipids bind to the anionic BEV surface, enabling hybrid nanoparticle formation. Nakase et al. utilized cationic lipids to fuse pH-sensitive peptides with MEVs, improving cytoplasmic delivery (347), while Sawada et al. engineered cationic pullulan-based nanogels to boost EV uptake efficiency (348). Receptor-ligand interactions further exemplify non-covalent precision. By exploiting natural binding pairs—such as transferrin (Tf) and its receptor (TfR)—Yang et al. isolated TfR + MEVs using magnetic nanoparticles, showcasing a strategy adaptable for BEV-specific targeting (349). Despite their simplicity, non-covalent methods may suffer from lower stability compared to covalent approaches, necessitating context-dependent optimization.

### 7.5 Administration strategies

Current BEV delivery approaches include oral, intravenous, and bioactive material-combined routes. Oral administration, though non-invasive, faces hurdles such as gastric acid degradation and intestinal variability. Surface modifications, such as dopamine polymerization, protect BEVs from acidic environments, enhancing gastric stability in pre-clinical settings (350).

Intravenous delivery, while potentially efficient, risks systemic toxicity; localized injections and hydrogel encapsulation are being developed as strategies toprolong retention and minimize off-target effects (351, 352). Integration with bioactive materials represents a promising experimental frontier in BEV applications. Liang Ma et al. combined nanotopographical titanium-cultured sEVs with 3D-printed polyetheretherketone scaffolds, significantly enhancing bone regeneration in rabbit femoral defects models (353). Similarly, hypoxia-responsive EVs (hypo-EVs) embedded in hydrogels promoted cranial defect repair in rats, leveraging the hypoxic bone microenvironment (354). These advances demonstrate the pre-clinical-stage synergistic potential of BEV-material hybrids in tissue engineering, necessitating future clinical validation.

### 7.6 Translational challenges and strategies for BEVs therapeutics

#### 7.6.1 Biosafety: Balancing efficacy and risk mitigation

The clinical advancement of bacterial extracellular vesicles (BEVs) hinges on rigorous biosafety validation. While pre-clinical studies confirm the absence of acute toxicity in BEV-administered models (355, 356), concerns persist regarding residual immunogenic components such as lipopolysaccharides (LPS). Strategies to enhance biocompatibility include genetic modifications targeting LPS biosynthesis (msbA, lpxM deletions) (357), leveraging non-pathogenic Gram-positive species or engineered probiotics (e.g., *Escherichia coli* Nissle 1917) (358), and physicochemical purification via lysozyme/pH treatments (359, 360). Encapsulation with pH-responsive biomaterials (e.g., calcium phosphate) further refines targeted delivery while minimizing systemic exposure (361). Comprehensive pharmacokinetic profiling—encompassing biodistribution, cellular uptake, and clearance mechanisms—remains critical to establish therapeutic windows and long-term safety thresholds.

#### 7.6.2 Standardization: toward reproducible BEV therapeutics

Heterogeneity in BEV production and isolation protocols poses significant reproducibility challenges. Variations in bacterial culture parameters (nutrient availability, pH, temperature) directly modulate vesicle yield and cargo composition (362, 363), while quantification methods based on protein content or nanoparticle counts introduce analytical inconsistencies (77, 364). Although commercial isolation kits improve accessibility, limitations in purity and cost-efficiency necessitate standardized workflows. Alignment with frameworks such as the MISEV guidelines (20, 365), coupled with advanced characterization tools (e.g., nanotracking analysis, proteomic profiling), could harmonize inter-laboratory practices and enhance data comparability.

#### 7.6.3 Scalability: bridging laboratory innovation to industrial feasibility

Fermentation technologies offer a scalable platform for BEV production, yet industrial translation faces bottlenecks. Heterologous protein expression in recombinant strains often requires suboptimal low-temperature induction (366, 367), conflicting with large-scale fermentation efficiency. Hypervesiculating mutants (e.g., nlpL, rmpM knockouts) (368, 369) and optimized bioreactor conditions (e.g., fed-batch strategies, dissolved oxygen control) (370, 371) present viable pathways to amplify vesicle yields. Future efforts must integrate synthetic biology with bioprocess engineering to achieve cost-effective, GMP-compliant manufacturing—a prerequisite for clinical adoption.

## 8 Conclusion and future perspectives

OP management faces persistent challenges with conventional therapies due to adverse effects and inadequate modulation of gut-bone crosstalk. NGPs and BEVs represent promising experimental transformative strategies that address these limitations through precision-targeted mechanisms. NGPs, enhanced by synthetic biology, hold potential to enable dynamic delivery of osteoprotective metabolites (e.g., SCFAs, phytoestrogens), immunomodulation, and neuroendocrine regulation, while circumventing colonization barriers inherent to traditional probiotics. BEVs, as biocompatible nanocarriers, offer experimental, scalable, non-replicative platforms for bone-specific delivery of therapeutic cargo (miRNAs, cytokines, osteogenic factors), synergizing with biomaterials to enhance fracture repair. Despite encouraging pre-clinical promise, significant challenges in strain-specific efficacy, biosafety, and industrial scalability necessitate standardized protocols and rigorous clinical validation. Future research must prioritize multi-omics integration to decode host-microbe interactions, optimize genetic toolkits for non-conventional probiotics, and advance hybrid BEV-scaffold systems. By bridging microbial therapeutics with bone bioengineering, these innovations hold immense potential to restore skeletal homeostasis, offering safer, personalized alternatives to reshape osteoporosis care.

Future directions should prioritize multifactorial strategies: Optimizing synthetic probiotics to enrich therapeutic strains, elucidating senescence-autophagy interplay in BMMSC aging, and advancing BEV engineering for dual osteoanabolic/anti-resorptive effects. Bridging these mechanistic insights with robust clinical validation is essential to unlock next-generation therapies, transforming OP management from symptomatic relief to pathophysiology-driven precision medicine.

## References

[1] Srivastava M Deal C. Osteoporosis in elderly: Prevention and treatment. *Clin Geriatr Med.* (2002) 18:529–55. 10.1016/s0749-0690(02)00022-8 12424871

[2] Gosset A Pouillès J Trémollieres F. Menopausal hormone therapy for the management of osteoporosis. *Best Pract Res Clin Endocrinol Metab.* (2021) 35:101551. 10.1016/j.beem.2021.101551 34119418

[3] Muñoz M Robinson K Shibli-Rahhal A. Bone health and osteoporosis prevention and treatment. *Clin Obstet Gynecol.* (2020) 63:770–87. 10.1097/GRF.0000000000000572 33017332

[4] Palmieri C Lam E Vigushin D Coombes R. Value of SERMs in postmenopausal women. *Lancet.* (2004) 363:1477–8. 10.1016/S0140-6736(04)16118-7 15121421

[5] Wang J Thingholm L Skiecevičienė J Rausch P Kummen M Hov J Genome-wide association analysis identifies variation in vitamin D receptor and other host factors influencing the gut microbiota. *Nat Genet.* (2016) 48:1396–406. 10.1038/ng.3695 27723756 PMC5626933

[6] Wang J Wu S Zhang Y Yang J Hu Z. Gut microbiota and calcium balance. *Front Microbiol.* (2022) 13:1033933. 10.3389/fmicb.2022.1033933 36713159 PMC9881461

[7] Pacifici R. Bone remodeling and the microbiome. *Cold Spring Harb Perspect Med.* (2018) 8:a031203. 10.1101/cshperspect.a031203 28847904 PMC5880157

[8] Wong C Yu J. Gut microbiota in colorectal cancer development and therapy. *Nat Rev Clin Oncol.* (2023) 20:429–52. 10.1038/s41571-023-00766-x 37169888

[9] Dziedzic A Saluk J. Probiotics and commensal gut microbiota as the effective alternative therapy for multiple sclerosis patients treatment. *Int J Mol Sci.* (2022) 23:14478. 10.3390/ijms232214478 36430954 PMC9699268

[10] Bungau S Behl T Singh A Sehgal A Singh S Chigurupati S Targeting probiotics in rheumatoid arthritis. *Nutrients.* (2021) 13:3376. 10.3390/nu13103376 34684377 PMC8539185

[11] Nilsson A Sundh D Bäckhed F Lorentzon M. Lactobacillus reuteri reduces bone loss in older women with low bone mineral density: A randomized, placebo-controlled, double-blind, clinical trial. *J Intern Med.* (2018) 284:307–17. 10.1111/joim.12805 29926979

[12] Yang L Lin S Li I Chen Y Tzu S Chou W Lactobacillus plantarum GKM3 and Lactobacillus paracasei GKS6 supplementation ameliorates bone loss in ovariectomized mice by promoting osteoblast differentiation and inhibiting osteoclast formation. *Nutrients.* (2020) 12:1914. 10.3390/nu12071914 32605314 PMC7401263

[13] Sapra L Shokeen N Porwal K Saini C Bhardwaj A Mathew M Bifidobacterium longum ameliorates ovariectomy-induced bone loss via enhancing anti-osteoclastogenic and immunomodulatory potential of regulatory B cells (Bregs). *Front Immunol.* (2022) 13:875788. 10.3389/fimmu.2022.875788 35693779 PMC9174515

[14] Liu J Chen C Liu Z Luo Z Rao S Jin L Extracellular vesicles from child gut microbiota enter into bone to preserve bone mass and strength. *Adv Sci (Weinh).* (2021) 8:2004831. 10.1002/advs.202004831 33977075 PMC8097336

[15] Suez J Zmora N Segal E Elinav E. The pros, cons, and many unknowns of probiotics. *Nat Med.* (2019) 25:716–29. 10.1038/s41591-019-0439-x 31061539

[16] Adolfsen K Callihan I Monahan C Greisen P Spoonamore J Momin M Improvement of a synthetic live bacterial therapeutic for phenylketonuria with biosensor-enabled enzyme engineering. *Nat Commun.* (2021) 12:6215. 10.1038/s41467-021-26524-0 34711827 PMC8553829

[17] Lubkowicz D Ho C Hwang I Yew W Lee Y Chang M. Reprogramming probiotic Lactobacillus reuteri as a biosensor for Staphylococcus aureus derived AIP-I detection. *ACS Synth Biol.* (2018) 7:1229–37. 10.1021/acssynbio.8b00063 29652493

[18] Perreault M Means J Gerson E Lee D Horvath M Rajasuriyar A *Activity of synb1353, an investigational methionine-consuming synthetic biotic medicine, in an acute nonhuman primate model of homocystinuria. *Mol Genet Metab.* (2022) 135:292–3. 10.1016/j.ymgme.2022.01.075.

[19] Riglar D Silver P. Engineering bacteria for diagnostic and therapeutic applications. *Nat Rev Microbiol.* (2018) 16:214–25. 10.1038/nrmicro.2017.172 29398705

[20] Witwer K Goberdhan D O’Driscoll L Théry C Welsh J Blenkiron C Updating MISEV: Evolving the minimal requirements for studies of extracellular vesicles. *J Extracell Vesicles.* (2021) 10:e12182. 10.1002/jev2.12182 34953156 PMC8710080

[21] Krishnan N Kubiatowicz L Holay M Zhou J Fang R Zhang L. Bacterial membrane vesicles for vaccine applications. *Adv Drug Deliv Rev.* (2022) 185:114294. 10.1016/j.addr.2022.114294 35436569

[22] Liu H Fang G Wu H Li Z Ye Q. L-Cysteine production in *Escherichia coli* based on rational metabolic engineering and modular strategy. *Biotechnol J.* (2018) 13:e1700695. 10.1002/biot.201700695 29405609

[23] Toyofuku M Nomura N Eberl L. Types and origins of bacterial membrane vesicles. *Nat Rev Microbiol.* (2019) 17:13–24. 10.1038/s41579-018-0112-2 30397270

[24] Hattner R Epker B Frost H. Suggested sequential mode of control of changes in cell behaviour in adult bone remodelling. *Nature.* (1965) 206:489–90. 10.1038/206489a0 5319106

[25] Eriksen E. Normal and pathological remodeling of human trabecular bone: Three dimensional reconstruction of the remodeling sequence in normals and in metabolic bone disease. *Endocr Rev.* (1986) 7:379–408. 10.1210/edrv-7-4-379 3536460

[26] Frost H. Skeletal structural adaptations to mechanical usage (SATMU): 1. Redefining Wolff’s law: The bone modeling problem. *Anat Rec.* (1990) 226:403–13. 10.1002/ar.1092260402 2184695

[27] Kobayashi S Takahashi H Ito A Saito N Nawata M Horiuchi H Trabecular minimodeling in human iliac bone. *Bone.* (2003) 32:163–9. 10.1016/s8756-3282(02)00947-x 12633788

[28] Boudin E Van Hul W. Mechanisms in endocrinology: Genetics of human bone formation. *Eur J Endocrinol.* (2017) 177:R69–83. 10.1530/EJE-16-0990 28381451

[29] Boivin G Farlay D Bala Y Doublier A Meunier P Delmas P. Influence of remodeling on the mineralization of bone tissue. *Osteoporos Int.* (2009) 20:1023–6. 10.1007/s00198-009-0861-x 19340504 PMC2904474

[30] Genant H Libanati C Engelke K Zanchetta J Høiseth A Yuen C Improvements in hip trabecular, subcortical, and cortical density and mass in postmenopausal women with osteoporosis treated with denosumab. *Bone.* (2013) 56:482–8. 10.1016/j.bone.2013.07.011 23871852

[31] Zebaze R Libanati C McClung M Zanchetta J Kendler D Høiseth A Denosumab reduces cortical porosity of the proximal femoral shaft in postmenopausal women with osteoporosis. *J Bone Miner Res.* (2016) 31:1827–34. 10.1002/jbmr.2855 27082709

[32] Dempster D Zhou H Ruff V Melby T Alam J Taylor K. Longitudinal effects of teriparatide or zoledronic acid on bone modeling- and remodeling-based formation in the SHOTZ study. *J Bone Miner Res.* (2018) 33:627–33. 10.1002/jbmr.3350 29194749

[33] Dempster D Zhou H Recker R Brown J Recknor C Lewiecki E Remodeling- and modeling-based bone formation with teriparatide versus denosumab: A longitudinal analysis from baseline to 3 months in the AVA study. *J Bone Miner Res.* (2018) 33:298–306. 10.1002/jbmr.3309 29024120

[34] McClung M Brown J Diez-Perez A Resch H Caminis J Meisner P Effects of 24 months of treatment with romosozumab followed by 12 months of denosumab or placebo in postmenopausal women with low bone mineral density: A randomized, double-blind, phase 2, parallel group study. *J Bone Miner Res.* (2018) 33:1397–406. 10.1002/jbmr.3452 29694685

[35] Ominsky M Niu Q Li C Li X Ke H. Tissue-level mechanisms responsible for the increase in bone formation and bone volume by sclerostin antibody. *J Bone Miner Res.* (2014) 29:1424–30. 10.1002/jbmr.2152 24967455

[36] Cadarette S Katz J Brookhart M Stürmer T Stedman M Levin R Comparative gastrointestinal safety of weekly oral bisphosphonates. *Osteoporos Int.* (2009) 20:1735–47. 10.1007/s00198-009-0871-8 19266138 PMC3257315

[37] Kanis J Reginster J Kaufman J Ringe J Adachi J Hiligsmann M A reappraisal of generic bisphosphonates in osteoporosis. *Osteoporos Int.* (2012) 23:213–21. 10.1007/s00198-011-1796-6 21953472 PMC3249199

[38] Adami S Bhalla A Dorizzi R Montesanti F Rosini S Salvagno G The acute-phase response after bisphosphonate administration. *Calcif Tissue Int.* (1987) 41:326–31. 10.1007/BF02556671 3124942

[39] Sauty A Pecherstorfer M Zimmer-Roth I Fioroni P Juillerat L Markert M Interleukin-6 and tumor necrosis factor alpha levels after bisphosphonates treatment in vitro and in patients with malignancy. *Bone.* (1996) 18:133–9. 10.1016/8756-3282(95)00448-3 8833207

[40] Black D Delmas P Eastell R Reid I Boonen S Cauley J Once-yearly zoledronic acid for treatment of postmenopausal osteoporosis. *N Engl J Med.* (2007) 356:1809–22. 10.1056/NEJMoa067312 17476007

[41] Heckbert S Li G Cummings S Smith N Psaty B. Use of alendronate and risk of incident atrial fibrillation in women. *Arch Intern Med.* (2008) 168:826–31. 10.1001/archinte.168.8.826 18443257

[42] Rhee C Lee J Oh S Choi N Park B. Use of bisphosphonate and risk of atrial fibrillation in older women with osteoporosis. *Osteoporos Int.* (2012) 23:247–54. 10.1007/s00198-011-1608-z 21431993

[43] Durnian J Olujohungbe A Kyle G. Bilateral acute uveitis and conjunctivitis after zoledronic acid therapy. *Eye (Lond).* (2005) 19:221–2. 10.1038/sj.eye.6701461 15258605

[44] Kaur H Uy C Kelly J Moses A. Orbital inflammatory disease in a patient treated with zoledronate. *Endocr Pract.* (2011) 17:e101–3. 10.4158/EP11081.CR 21803720

[45] Patel D Horne A House M Reid I McGhee C. The incidence of acute anterior uveitis after intravenous zoledronate. *Ophthalmology.* (2013) 120:773–6. 10.1016/j.ophtha.2012.10.028 23290982

[46] Park-Wyllie L Mamdani M Juurlink D Hawker G Gunraj N Austin P Bisphosphonate use and the risk of subtrochanteric or femoral shaft fractures in older women. *JAMA.* (2011) 305:783–9. 10.1001/jama.2011.190 21343577

[47] Hsiao F Huang W Chen Y Wen Y Kao Y Chen L Hip and subtrochanteric or diaphyseal femoral fractures in alendronate users: A 10-year, nationwide retrospective cohort study in Taiwanese women. *Clin Ther.* (2011) 33:1659–67. 10.1016/j.clinthera.2011.09.006 22018450

[48] Mahjoub Z Jean S Leclerc J Brown J Boulet D Pelet S Incidence and characteristics of atypical femoral fractures: Clinical and geometrical data. *J Bone Miner Res.* (2016) 31:767–76. 10.1002/jbmr.2748 26588590

[49] Mavrokokki T Cheng A Stein B Goss A. Nature and frequency of bisphosphonate-associated osteonecrosis of the jaws in Australia. *J Oral Maxillofac Surg.* (2007) 65:415–23. 10.1016/j.joms.2006.10.061 17307586

[50] Khan A Rios L Sándor G Khan N Peters E Rahman M Bisphosphonate-associated osteonecrosis of the jaw in Ontario: A survey of oral and maxillofacial surgeons. *J Rheumatol.* (2011) 38:1396–402. 10.3899/jrheum.100221 21498483

[51] Tennis P Rothman K Bohn R Tan H Zavras A Laskarides C Incidence of osteonecrosis of the jaw among users of bisphosphonates with selected cancers or osteoporosis. *Pharmacoepidemiol Drug Saf.* (2012) 21:810–7. 10.1002/pds.3292 22711458

[52] Cartsos V Zhu S Zavras A. Bisphosphonate use and the risk of adverse jaw outcomes: A medical claims study of 714,217 people. *J Am Dent Assoc.* (2008) 139:23–30. 10.14219/jada.archive.2008.0016 18167381

[53] Sedghizadeh P Kumar S Gorur A Schaudinn C Shuler C Costerton J. Microbial biofilms in osteomyelitis of the jaw and osteonecrosis of the jaw secondary to bisphosphonate therapy. *J Am Dent Assoc.* (2009) 140:1259–65. 10.14219/jada.archive.2009.0049 19797556

[54] Lesclous P Abi Najm S Carrel J Baroukh B Lombardi T Willi J Bisphosphonate-associated osteonecrosis of the jaw: A key role of inflammation? *Bone.* (2009) 45:843–52. 10.1016/j.bone.2009.07.011 19631301

[55] Santini D Vincenzi B Dicuonzo G Avvisati G Massacesi C Battistoni F *Zoledronic acid induces significant and long-lasting modifications of circulating angiogenic factors in cancer patients. *Clin Cancer Res.* (2003) 9:2893–7. 12912933

[56] Fournier P Boissier S Filleur S Guglielmi J Cabon F Colombel M Bisphosphonates inhibit angiogenesis in vitro and testosterone-stimulated vascular regrowth in the ventral prostate in castrated rats. *Cancer Res.* (2002) 62:6538–44. 12438248

[57] Diédhiou D Cuny T Sarr A Norou Diop S Klein M Weryha G. Efficacy and safety of denosumab for the treatment of osteoporosis: A systematic review. *Ann Endocrinol (Paris).* (2015) 76:650–7. 10.1016/j.ando.2015.10.009 26639186

[58] Neer R Arnaud C Zanchetta J Prince R Gaich G Reginster J Effect of parathyroid hormone (1-34) on fractures and bone mineral density in postmenopausal women with osteoporosis. *N Engl J Med.* (2001) 344:1434–41. 10.1056/NEJM200105103441904 11346808

[59] Andrews E Gilsenan A Midkiff K Sherrill B Wu Y Mann B The US postmarketing surveillance study of adult osteosarcoma and teriparatide: Study design and findings from the first 7 years. *J Bone Miner Res.* (2012) 27:2429–37. 10.1002/jbmr.1768 22991313 PMC3546381

[60] Delmas P Ensrud K Adachi J Harper K Sarkar S Gennari C Efficacy of raloxifene on vertebral fracture risk reduction in postmenopausal women with osteoporosis: Four-year results from a randomized clinical trial. *J Clin Endocrinol Metab.* (2002) 87:3609–17. 10.1210/jcem.87.8.8750 12161484

[61] Vogel V Costantino J Wickerham D Cronin W Cecchini R Atkins J Effects of tamoxifen vs raloxifene on the risk of developing invasive breast cancer and other disease outcomes: The NSABP study of tamoxifen and raloxifene (STAR) P-2 trial. *JAMA.* (2006) 295:2727–41. 10.1001/jama.295.23.joc60074 16754727

[62] Marcano A Taormina D Egol K Peck V Tejwani N. Are race and sex associated with the occurrence of atypical femoral fractures? *Clin Orthop Relat Res.* (2014) 472:1020–7. 10.1007/s11999-013-3352-5 24166075 PMC3916602

[63] Khan A Morrison A Hanley D Felsenberg D McCauley L O’Ryan F Diagnosis and management of osteonecrosis of the jaw: A systematic review and international consensus. *J Bone Miner Res.* (2015) 30:3–23. 10.1002/jbmr.2405 25414052

[64] Sjögren K Engdahl C Henning P Lerner U Tremaroli V Lagerquist M The gut microbiota regulates bone mass in mice. *J Bone Miner Res.* (2012) 27:1357–67. 10.1002/jbmr.1588 22407806 PMC3415623

[65] Tyagi A Yu M Darby T Vaccaro C Li J Owens J The microbial metabolite butyrate stimulates bone formation via T regulatory cell-mediated regulation of WNT10B expression. *Immunity.* (2018) 49:1116–31.e7. 10.1016/j.immuni.2018.10.013. 30446387 PMC6345170

[66] D’Amelio P Sassi F. Osteoimmunology: From mice to humans. *Bonekey Rep.* (2016) 5:802. 10.1038/bonekey.2016.29 27195109 PMC4870940

[67] Li J Yu M Pal S Tyagi A Dar H Adams J Parathyroid hormone-dependent bone formation requires butyrate production by intestinal microbiota. *J Clin Invest.* (2020) 130:1767–81. 10.1172/JCI133473 31917685 PMC7108906

[68] Yu M Malik Tyagi A Li J Adams J Denning T Weitzmann M PTH induces bone loss via microbial-dependent expansion of intestinal TNF+ T cells and Th17 cells. *Nat Commun.* (2020) 11:468. 10.1038/s41467-019-14148-4 31980603 PMC6981196

[69] Zhang B Hou R Zou Z Luo T Zhang Y Wang L Mechanically induced autophagy is associated with ATP metabolism and cellular viability in osteocytes in vitro. *Redox Biol.* (2018) 14:492–8. 10.1016/j.redox.2017.10.021 29096322 PMC5680519

[70] Lin N Chen C Kagwiria R Liang R Beyer C Distler A Inactivation of autophagy ameliorates glucocorticoid-induced and ovariectomy-induced bone loss. *Ann Rheum Dis.* (2016) 75:1203–10. 10.1136/annrheumdis-2015-207240 26113650

[71] Ma Y Qi M An Y Zhang L Yang R Doro D Autophagy controls mesenchymal stem cell properties and senescence during bone aging. *Aging Cell.* (2018) 17:e12709. 10.1111/acel.12709 29210174 PMC5770781

[72] Guo Y Jia X Cui Y Song Y Wang S Geng Y Sirt3-mediated mitophagy regulates AGEs-induced BMSCs senescence and senile osteoporosis. *Redox Biol.* (2021) 41:101915. 10.1016/j.redox.2021.101915 33662874 PMC7930642

[73] Farr J Fraser D Wang H Jaehn K Ogrodnik M Weivoda M Identification of senescent cells in the bone microenvironment. *J Bone Miner Res.* (2016) 31:1920–9. 10.1002/jbmr.2892 27341653 PMC5289710

[74] Farr J Xu M Weivoda M Monroe D Fraser D Onken J Targeting cellular senescence prevents age-related bone loss in mice. *Nat Med.* (2017) 23:1072–9. 10.1038/nm.4385 28825716 PMC5657592

[75] Yi L Ju Y He Y Yin X Xu Y Weng T. Intraperitoneal injection of Desferal^®^ alleviated the age-related bone loss and senescence of bone marrow stromal cells in rats. *Stem Cell Res Ther.* (2021) 12:45. 10.1186/s13287-020-02112-9 33413663 PMC7791659

[76] Hickson L Langhi Prata L Bobart S Evans T Giorgadze N Hashmi S Senolytics decrease senescent cells in humans: Preliminary report from a clinical trial of Dasatinib plus Quercetin in individuals with diabetic kidney disease. *EBioMedicine.* (2019) 47:446–56. 10.1016/j.ebiom.2019.08.069 31542391 PMC6796530

[77] Chen C Rao S Yue T Tan Y Yin H Chen L Glucocorticoid-induced loss of beneficial gut bacterial extracellular vesicles is associated with the pathogenesis of osteonecrosis. *Sci Adv.* (2022) 8:eabg8335. 10.1126/sciadv.abg8335 35417243 PMC9007505

[78] Sanders M Merenstein D Reid G Gibson G Rastall R. Probiotics and prebiotics in intestinal health and disease: From biology to the clinic. *Nat Rev Gastroenterol Hepatol.* (2019) 16:605–16. 10.1038/s41575-019-0173-3 31296969

[79] Kujawska M Neuhaus K Huptas C Jiménez E Arboleya S Schaubeck M Exploring the potential probiotic properties of Bifidobacterium breve DSM 32583-A novel strain isolated from human milk. *Probiotics Antimicrob Proteins.* (2024): 10.1007/s12602-024-10346-9 39287748 PMC12634702

[80] Khalighi A Behdani R Kouhestani S. *Probiotics: A Comprehensive Review of Their Classification, Mode of Action and Role in Human Nutrition.* London: Intechopen (2016). 10.5772/63646

[81] Maftei N Raileanu C Balta A Ambrose L Boev M Marin D The potential impact of probiotics on human health: An update on their health-promoting properties. *Microorganisms.* (2024) 12:234. 10.3390/microorganisms12020234 38399637 PMC10891645

[82] Vera-Santander V Hernández-Figueroa R Jiménez-Munguía M Mani-López E López-Malo A. Health benefits of consuming foods with bacterial probiotics, postbiotics, and their metabolites: A review. *Molecules.* (2023) 28:1230. 10.3390/molecules28031230 36770898 PMC9920731

[83] Serek P Oleksy-Wawrzyniak M. The effect of bacterial infections, probiotics and zonulin on intestinal barrier integrity. *Int J Mol Sci.* (2021) 22:11359. 10.3390/ijms222111359 34768787 PMC8583036

[84] Rose E Odle J Blikslager A Ziegler A. Probiotics, prebiotics and epithelial tight junctions: A promising approach to modulate intestinal barrier function. *Int J Mol Sci.* (2021) 22:6729. 10.3390/ijms22136729 34201613 PMC8268081

[85] Corsetti A Settanni L. Lactobacilli in sourdough fermentation. *Food Res Int.* (2007) 40:539–58. 10.1016/j.foodres.2006.11.001

[86] Yan F Li N Shi J Li H Yue Y Jiao W Lactobacillus acidophilus alleviates type 2 diabetes by regulating hepatic glucose, lipid metabolism and gut microbiota in mice. *Food Funct.* (2019) 10:5804–15. 10.1039/c9fo01062a 31461095

[87] Mu Q Tavella V Luo X. Role of Lactobacillus reuteri in human health and diseases. *Front Microbiol.* (2018) 9:757. 10.3389/fmicb.2018.00757 29725324 PMC5917019

[88] Zhan H He Y Wang Q Liu Q He L Tao L Evaluation of probiotic strains isolated from artemisia argyi fermentation liquor and the antagonistic effect of Lactiplantibacillus plantarum against pathogens. *Fermentation.* (2023) 9:536. 10.3390/fermentation9060536

[89] Lee J Kim J Choi J Noh B Kim H Cho E. Anti-Inflammatory effects of Artemisia argyi H. fermented by Lactobacillus plantarum in the LPS-induced RAW 264.7 cells and DSS-induced colitis model. *Foods.* (2024) 13:998. 10.3390/foods13070998 38611304 PMC11011819

[90] Fusco W Lorenzo M Cintoni M Porcari S Rinninella E Kaitsas F Short-chain fatty-acid-producing bacteria: Key components of the human gut microbiota. *Nutrients.* (2023) 15:2211. 10.3390/nu15092211 37432351 PMC10180739

[91] Lavelle A Sokol H. Gut microbiota-derived metabolites as key actors in inflammatory bowel disease. *Nat Rev Gastroenterol Hepatol.* (2020) 17:223–37. 10.1038/s41575-019-0258-z 32076145

[92] Di Giacomo S Toussaint F Ledesma-García L Knoops A Vande Capelle F Fremaux C Expanding natural transformation to improve beneficial lactic acid bacteria. *FEMS Microbiol Rev.* (2022) 46:fuac014. 10.1093/femsre/fuac014 35254446 PMC9300618

[93] O’Sullivan D Klaenhammer T. High- and low-copy-number Lactococcus shuttle cloning vectors with features for clone screening. *Gene.* (1993) 137:227–31. 10.1016/0378-1119(93)90011-q 8299952

[94] Bao S Zhu L Zhuang Q Wang L Xu P Itoh K Distribution dynamics of recombinant Lactobacillus in the gastrointestinal tract of neonatal rats. *PLoS One.* (2013) 8:e60007. 10.1371/journal.pone.0060007 23544119 PMC3609735

[95] Walker D Klaenhammer T. Isolation of a novel IS3 group insertion element and construction of an integration vector for Lactobacillus spp. *J Bacteriol.* (1994) 176:5330–40. 10.1128/jb.176.17.5330-5340.1994 8071209 PMC196718

[96] Russell W Klaenhammer T. Efficient system for directed integration into the Lactobacillus acidophilus and Lactobacillus gasseri chromosomes via homologous recombination. *Appl Environ Microbiol.* (2001) 67:4361–4. 10.1128/AEM.67.9.4361-4364.2001 11526048 PMC93172

[97] Song A Palmiter R. Detecting and avoiding problems when using the cre-lox system. *Trends Genet.* (2018) 34:333–40. 10.1016/j.tig.2017.12.008 29336844 PMC5910175

[98] Oh J van Pijkeren JP. CRISPR-Cas9-assisted recombineering in Lactobacillus reuteri. *Nucleic Acids Res.* (2014) 42:e131. 10.1093/nar/gku623 25074379 PMC4176153

[99] van der Els S James J Kleerebezem M Bron P. Versatile Cas9-driven subpopulation selection toolbox for Lactococcus lactis. *Appl Environ Microbiol.* (2018) 84:e2752–2717. 10.1128/AEM.02752-17 29453254 PMC5881059

[100] Mierau I Kleerebezem M. 10 years of the nisin-controlled gene expression system (NICE) in Lactococcus lactis. *Appl Microbiol Biotechnol.* (2005) 68:705–17. 10.1007/s00253-005-0107-6 16088349

[101] Berlec A Škrlec K Kocjan J Olenic M Štrukelj B. Single plasmid systems for inducible dual protein expression and for CRISPR-Cas9/CRISPRi gene regulation in lactic acid bacterium Lactococcus lactis. *Sci Rep.* (2018) 8:1009. 10.1038/s41598-018-19402-1 29343791 PMC5772564

[102] Hidalgo-Cantabrana C O’Flaherty S Barrangou R. CRISPR-based engineering of next-generation lactic acid bacteria. *Curr Opin Microbiol.* (2017) 37:79–87. 10.1016/j.mib.2017.05.015 28622636

[103] Kieliszek M Pobiega K Piwowarek K Kot A. Characteristics of the proteolytic enzymes produced by lactic acid bacteria. *Molecules.* (2021) 26:1858. 10.3390/molecules26071858 33806095 PMC8037685

[104] Parvaneh M Karimi G Jamaluddin R Ng M Zuriati I Muhammad S. Lactobacillus helveticus (ATCC 27558) upregulates Runx2 and Bmp2 and modulates bone mineral density in ovariectomy-induced bone loss rats. *Clin Interv Aging.* (2018) 13:1555–64. 10.2147/CIA.S169223 30214175 PMC6121767

[105] Parvaneh K Ebrahimi M Sabran M Karimi G Hwei A Abdul-Majeed S Probiotics (Bifidobacterium longum) increase bone mass density and upregulate sparc and Bmp-2 genes in rats with bone loss resulting from ovariectomy. *Biomed Res Int.* (2015) 2015:897639. 10.1155/2015/897639 26366421 PMC4558422

[106] Li S Han X Liu N Chang J Liu G Hu S. Lactobacillus plantarum attenuates glucocorticoid-induced osteoporosis by altering the composition of rat gut microbiota and serum metabolic profile. *Front Immunol.* (2023) 14:1285442. 10.3389/fimmu.2023.1285442 38264658 PMC10803555

[107] Raveschot C Coutte F Frémont M Vaeremans M Dugersuren J Demberel S Probiotic Lactobacillus strains from Mongolia improve calcium transport and uptake by intestinal cells in vitro. *Food Res Int.* (2020) 133:109201. 10.1016/j.foodres.2020.109201 32466902

[108] Du R Jiao S Dai Y An J Lv J Yan X Probiotic Bacillus amyloliquefaciens C-1 improves growth performance, stimulates GH/IGF-1, and regulates the gut microbiota of growth-retarded beef calves. *Front Microbiol.* (2018) 9:2006. 10.3389/fmicb.2018.02006 30210477 PMC6120984

[109] Leong T Lo W Lee W Tan B Lee X Lee L Leveraging advances in immunopathology and artificial intelligence to analyze in vitro tumor models in composition and space. *Adv Drug Deliv Rev.* (2021) 177:113959. 10.1016/j.addr.2021.113959 34481035

[110] Reel P Reel S Pearson E Trucco E Jefferson E. Using machine learning approaches for multi-omics data analysis: A review. *Biotechnol Adv.* (2021) 49:107739. 10.1016/j.biotechadv.2021.107739 33794304

[111] Westfall S Carracci F Estill M Zhao D Wu Q Shen L Optimization of probiotic therapeutics using machine learning in an artificial human gastrointestinal tract. *Sci Rep.* (2021) 11:1067. 10.1038/s41598-020-79947-y 33441743 PMC7806704

[112] Zampieri G Efthimiou G Angione C. Multi-dimensional experimental and computational exploration of metabolism pinpoints complex probiotic interactions. *Metab Eng.* (2023) 76:120–32. 10.1016/j.ymben.2023.01.008 36720400

[113] McCoubrey L Seegobin N Elbadawi M Hu Y Orlu M Gaisford S Active Machine learning for formulation of precision probiotics. *Int J Pharm.* (2022) 616:121568. 10.1016/j.ijpharm.2022.121568 35150845

[114] Bergamini C Bianchi N Giaccone V Catellani P Alberghini L Stella A Machine learning algorithms highlight tRNA information content and chargaff’s second parity rule score as important features in discriminating probiotics from non-probiotics. *Biology (Basel).* (2022) 11:1024. 10.3390/biology11071024 36101405 PMC9311688

[115] Padmavathi T Bhargavi R Priyanka P Niranjan N Pavitra P. Screening of potential probiotic lactic acid bacteria and production of amylase and its partial purification. *J Genet Eng Biotechnol.* (2018) 16:357–62. 10.1016/j.jgeb.2018.03.005 30733746 PMC6353751

[116] Kobayashi S Shieh J Ruiz de Sabando A Kim J Liu Y Zee SY Deep learning-based approach to the characterization and quantification of histopathology in mouse models of colitis. *PLoS One.* (2022) 17:e0268954. 10.1371/journal.pone.0268954 36037173 PMC9423669

[117] Arlova A Jin C Wong-Rolle A Chen E Lisle C Brown G Artificial intelligence-based tumor segmentation in mouse models of lung adenocarcinoma. *J Pathol Inform.* (2022) 13:100007. 10.1016/j.jpi.2022.100007 35242446 PMC8860735

[118] Piewngam P Otto M. Bacillus subtilis application on decolonisation of Staphylococcus aureus - Authors’ reply. *Lancet Microbe.* (2023) 4:e393. 10.1016/S2666-5247(23)00066-6 36893779

[119] Savytska M Kyriienko D Komisarenko I Kovalchuk O Falalyeyeva T Kobyliak N. Probiotic for pancreatic β-Cell function in type 2 diabetes: A randomized, double-blinded, placebo-controlled clinical trial. *Diabetes Ther.* (2023) 14:1915–31. 10.1007/s13300-023-01474-6 37713103 PMC10570251

[120] An H Douillard F Wang G Zhai Z Yang J Song S Integrated transcriptomic and proteomic analysis of the bile stress response in a centenarian-originated probiotic Bifidobacterium longum BBMN68. *Mol Cell Proteomics.* (2014) 13:2558–72. 10.1074/mcp.M114.039156 24965555 PMC4188986

[121] Xu H Wu L Pan D Zeng X Cai Z Guo Y Adhesion characteristics and dual transcriptomic and proteomic analysis of Lactobacillus reuteri SH23 upon gastrointestinal fluid stress. *J Proteome Res.* (2021) 20:2447–57. 10.1021/acs.jproteome.0c00933 33705137

[122] Palmer J Piattelli E McCormick B Silby M Brigham C Bucci V. Engineered Probiotic for the Inhibition of *Salmonella* via tetrathionate-induced production of Microcin H47. *ACS Infect Dis.* (2018) 4:39–45. 10.1021/acsinfecdis.7b00114 28918634 PMC5766358

[123] Yan F Cao H Cover T Whitehead R Washington M Polk D. Soluble proteins produced by probiotic bacteria regulate intestinal epithelial cell survival and growth. *Gastroenterology.* (2007) 132:562–75. 10.1053/j.gastro.2006.11.022 17258729 PMC3036990

[124] Blaak E Canfora E Theis S Frost G Groen A Mithieux G Short chain fatty acids in human gut and metabolic health. *Benef Microbes.* (2020) 11:411–55. 10.3920/BM2020.0057 32865024

[125] Zheng P Fang H Yang H Tien N Wang M Wu J. Lactobacillus pentosus strain LPS16 produces lactic acid, inhibiting multidrug-resistant *Helicobacter* pylori. *J Microbiol Immunol Infect.* (2016) 49:168–74. 10.1016/j.jmii.2014.04.014 24874430

[126] Cammarota G Ianiro G Ahern A Carbone C Temko A Claesson M Gut microbiome, big data and machine learning to promote precision medicine for cancer. *Nat Rev Gastroenterol Hepatol.* (2020) 17:635–48. 10.1038/s41575-020-0327-3 32647386

[127] Feng S Sterzenbach R Guo X. Deep learning for peptide identification from metaproteomics datasets. *J Proteomics.* (2021) 247:104316. 10.1016/j.jprot.2021.104316 34246788 PMC8435027

[128] Reiman D Dai Y. Using Autoencoders for predicting latent microbiome community shifts responding to dietary changes. In *Proceedings of the 2019 IEEE International Conference on Bioinformatics and Biomedicine (BIBM).* Piscataway, NJ: IEEE (2019).

[129] Sharma D Paterson A Xu W. TaxoNN: Ensemble of neural networks on stratified microbiome data for disease prediction. *Bioinformatics.* (2020) 36:4544–50. 10.1093/bioinformatics/btaa542 32449747 PMC7750934

[130] Reid G Gaudier E Guarner F Huffnagle G Macklaim J Munoz A Responders and non-responders to probiotic interventions: How can we improve the odds? *Gut Microbes.* (2010) 1:200–4. 10.4161/gmic.1.3.12013 21637034 PMC3023600

[131] Seymour C Gomez H Chang C Clermont G Kellum J Kennedy J Precision medicine for all? Challenges and opportunities for a precision medicine approach to critical illness. *Crit Care.* (2017) 21:257. 10.1186/s13054-017-1836-5 29047353 PMC5648512

[132] Zmora N Soffer E Elinav E. Transforming medicine with the microbiome. *Sci Transl Med.* (2019) 11:eaaw1815. 10.1126/scitranslmed.aaw1815 30700573

[133] Feng X McDonald J. Disorders of bone remodeling. *Annu Rev Pathol.* (2011) 6:121–45. 10.1146/annurev-pathol-011110-130203 20936937 PMC3571087

[134] Zaiss M Jones R Schett G Pacifici R. The gut-bone axis: How bacterial metabolites bridge the distance. *J Clin Invest.* (2019) 129:3018–28. 10.1172/JCI128521 31305265 PMC6668676

[135] Desfita S Sari W Yusmarini Y Pato U Zakłos-Szyda M Budryn G. Effect of fermented soymilk-honey from different probiotics on osteocalcin level in menopausal women. *Nutrients.* (2021) 13:3581. 10.3390/nu13103581 34684581 PMC8541044

[136] Zelante T Iannitti R Cunha C De Luca A Giovannini G Pieraccini G Tryptophan catabolites from microbiota engage aryl hydrocarbon receptor and balance mucosal reactivity via interleukin-22. *Immunity.* (2013) 39:372–85. 10.1016/j.immuni.2013.08.003 23973224

[137] Yan J Herzog J Tsang K Brennan C Bower M Garrett W Gut microbiota induce IGF-1 and promote bone formation and growth. *Proc Natl Acad Sci U S A.* (2016) 113:E7554–63. 10.1073/pnas.1607235113 27821775 PMC5127374

[138] Liu Y Yang R Liu X Zhou Y Qu C Kikuiri T Hydrogen sulfide maintains mesenchymal stem cell function and bone homeostasis via regulation of Ca(2+) channel sulfhydration. *Cell Stem Cell.* (2014) 15:66–78. 10.1016/j.stem.2014.03.005 24726192 PMC4082757

[139] Wan Y. Bone marrow mesenchymal stem cells: Fat on and blast off by FGF21. *Int J Biochem Cell Biol.* (2013) 45:546–9. 10.1016/j.biocel.2012.12.014 23270727 PMC3568182

[140] Tu Y Yang R Xu X Zhou X. The microbiota-gut-bone axis and bone health. *J Leukoc Biol.* (2021) 110:525–37. 10.1002/JLB.3MR0321-755R 33884666

[141] Gomez de Agüero M Ganal-Vonarburg SC Fuhrer T Rupp S Uchimura Y Li H The maternal microbiota drives early postnatal innate immune development. *Science.* (2016) 351:1296–302. 10.1126/science.aad2571 26989247

[142] Duscha A Hegelmaier T Dürholz K Desel C Gold R Zaiss M Propionic acid beneficially modifies osteoporosis biomarkers in patients with multiple sclerosis. *Ther Adv Neurol Disord.* (2022) 15:17562864221103935. 10.1177/17562864221103935 35755968 PMC9218497

[143] Guan Z Jia J Zhang C Sun T Zhang W Yuan W Gut microbiome dysbiosis alleviates the progression of osteoarthritis in mice. *Clin Sci (Lond).* (2020) 134:3159–74. 10.1042/CS20201224 33215637

[144] Patra S Sahu N Saxena S Pradhan B Nayak S Roychowdhury A. Effects of probiotics at the interface of metabolism and immunity to prevent colorectal cancer-associated gut inflammation: A systematic network and meta-analysis with molecular docking studies. *Front Microbiol.* (2022) 13:878297. 10.3389/fmicb.2022.878297 35711771 PMC9195627

[145] Plaza-Díaz J Ruiz-Ojeda F Vilchez-Padial L Gil A. Evidence of the anti-inflammatory effects of probiotics and synbiotics in intestinal chronic diseases. *Nutrients.* (2017) 9:555. 10.3390/nu9060555 28555037 PMC5490534

[146] Kekkonen R Lummela N Karjalainen H Latvala S Tynkkynen S Jarvenpaa S Probiotic intervention has strain-specific anti-inflammatory effects in healthy adults. *World J Gastroenterol.* (2008) 14:2029–36. 10.3748/wjg.14.2029 18395902 PMC2701523

[147] Schiavi E Gleinser M Molloy E Groeger D Frei R Ferstl R The surface-associated exopolysaccharide of Bifidobacterium longum 35624 plays an essential role in dampening host proinflammatory responses and repressing local TH17 responses. *Appl Environ Microbiol.* (2016) 82:7185–96. 10.1128/AEM.02238-16 27736791 PMC5118929

[148] Parker A Romano S Ansorge R Aboelnour A Le Gall G Savva G Fecal microbiota transfer between young and aged mice reverses hallmarks of the aging gut, eye, and brain. *Microbiome.* (2022) 10:68. 10.1186/s40168-022-01243-w 35501923 PMC9063061

[149] Simpkins J Yang S Sarkar S Pearce V. Estrogen actions on mitochondria–physiological and pathological implications. *Mol Cell Endocrinol.* (2008) 290:51–9. 10.1016/j.mce.2008.04.013 18571833 PMC2737506

[150] Marcucci G Domazetovic V Nediani C Ruzzolini J Favre C Brandi M. Oxidative stress and natural antioxidants in osteoporosis: Novel preventive and therapeutic approaches. *Antioxidants (Basel).* (2023) 12:373. 10.3390/antiox12020373 36829932 PMC9952369

[151] Manolagas S. From estrogen-centric to aging and oxidative stress: A revised perspective of the pathogenesis of osteoporosis. *Endocr Rev.* (2010) 31:266–300. 10.1210/er.2009-0024 20051526 PMC3365845

[152] Yuan Y Yang J Zhuge A Li L Ni S. Gut microbiota modulates osteoclast glutathione synthesis and mitochondrial biogenesis in mice subjected to ovariectomy. *Cell Prolif.* (2022) 55:e13194. 10.1111/cpr.13194 35080066 PMC8891549

[153] Iglesias-Aguirre C González-Sarrías A Cortés-Martín A Romo-Vaquero M Osuna-Galisteo L Cerón J In vivo administration of gut bacterial consortia replicates urolithin metabotypes A and B in a non-urolithin-producing rat model. *Food Funct.* (2023) 14:2657–67. 10.1039/d2fo03957e 36866688

[154] Wu F Peng Y Wang C Liu F. FRI0429 UROLITHIN B ATTENUATES The INFLAMMATORY AND NITROSATIVE STRESS ON INTERLEUKIN-1 INDUCED CHONDROCYTES. *Ann Rheumatic Dis.* (2020) 79:813.1–813. 10.1136/annrheumdis-2020-eular.5133

[155] Tao H Li W Zhang W Yang C Zhang C Liang X Urolithin A suppresses RANKL-induced osteoclastogenesis and postmenopausal osteoporosis by, suppresses inflammation and downstream NF-κB activated pyroptosis pathways. *Pharmacol Res.* (2021) 174:105967. 10.1016/j.phrs.2021.105967 34740817

[156] Osadchiy V Martin C Mayer E. The gut-brain axis and the microbiome: Mechanisms and clinical implications. *Clin Gastroenterol Hepatol.* (2019) 17:322–32. 10.1016/j.cgh.2018.10.002 30292888 PMC6999848

[157] Hanscom M Loane D Shea-Donohue T. Brain-gut axis dysfunction in the pathogenesis of traumatic brain injury. *J Clin Invest.* (2021) 131:e143777. 10.1172/JCI143777 34128471 PMC8203445

[158] Mao Q Shi L Wang Z Luo Y Wang Y Li X Chemical profiles and pharmacological activities of Chang-Kang-Fang, a multi-herb Chinese medicinal formula, for treating irritable bowel syndrome. *J Ethnopharmacol.* (2017) 201:123–35. 10.1016/j.jep.2017.02.045 28263849

[159] Sun J Wu X Meng Y Cheng J Ning H Peng Y Electro-acupuncture decreases 5-HT, CGRP and increases NPY in the brain-gut axis in two rat models of Diarrhea-predominant irritable bowel syndrome(D-IBS). *BMC Complement Altern Med.* (2015) 15:340. 10.1186/s12906-015-0863-5 26419631 PMC4589130

[160] Mittal R Debs L Patel A Nguyen D Patel K O’Connor G Neurotransmitters: The critical modulators regulating gut-brain axis. *J Cell Physiol.* (2017) 232:2359–72. 10.1002/jcp.25518 27512962 PMC5772764

[161] Wu Y Wang Y Hu A Shu X Huang W Liu J Lactobacillus plantarum-derived postbiotics prevent *Salmonella*-induced neurological dysfunctions by modulating gut-brain axis in mice. *Front Nutr.* (2022) 9:946096. 10.3389/fnut.2022.946096 35967771 PMC9365972

[162] Madabushi J Khurana P Gupta N Gupta M. Gut biome and mental health: Do probiotics work? *Cureus.* (2023) 15:e40293. 10.7759/cureus.40293 37448433 PMC10337499

[163] Zhang Y Cao M Li Y Dai G Lu P Zhang M The regulative effect and repercussion of probiotics and prebiotics on osteoporosis: Involvement of brain-gut-bone axis. *Crit Rev Food Sci Nutr.* (2022) 63:7510–28. 10.1080/10408398.2022.2047005 35234534

[164] Chudzik A Orzyłowska A Rola R Stanisz G. Probiotics, prebiotics and postbiotics on mitigation of depression symptoms: Modulation of the brain-gut-microbiome axis. *Biomolecules.* (2021) 11:1000. 10.3390/biom11071000 34356624 PMC8301955

[165] Bharwani A Bala A Surette M Bienenstock J Vigod S Taylor V. Gut microbiome patterns associated with treatment response in patients with major depressive disorder. *Can J Psychiatry.* (2020) 65:278–80. 10.1177/0706743719900464 31958990 PMC7385422

[166] Bravo J Forsythe P Chew M Escaravage E Savignac H Dinan T Ingestion of Lactobacillus strain regulates emotional behavior and central GABA receptor expression in a mouse via the vagus nerve. *Proc Natl Acad Sci U S A.* (2011) 108:16050–5. 10.1073/pnas.1102999108 21876150 PMC3179073

[167] Gareau M Jury J MacQueen G Sherman P Perdue M. Probiotic treatment of rat pups normalises corticosterone release and ameliorates colonic dysfunction induced by maternal separation. *Gut.* (2007) 56:1522–8. 10.1136/gut.2006.117176 17339238 PMC2095679

[168] Tatsuoka M Osaki Y Ohsaka F Tsuruta T Kadota Y Tochio T Consumption of indigestible saccharides and administration of Bifidobacterium pseudolongum reduce mucosal serotonin in murine colonic mucosa. *Br J Nutr.* (2022) 127:513–25. 10.1017/S0007114521001306 33849681

[169] Liu Y Dou S Li M Wang X. Production of indole and hydrogen sulfide by the oxygen-tolerant mutant strain Clostridium sp. Aeroto-AUH-JLC108 contributes to form a hypoxic microenvironment. *Arch Microbiol.* (2022) 204:486. 10.1007/s00203-022-03113-3 35834134

[170] Li G Young KD. A new suite of tnaA mutants suggests that *Escherichia coli* tryptophanase is regulated by intracellular sequestration and by occlusion of its active site. *BMC Microbiol.* (2015) 15:14. 10.1186/s12866-015-0346-3 25650045 PMC4323232

[171] Yan L Wang X Yu T Qi Z Li H Nan H Characteristics of the gut microbiota and serum metabolites in postmenopausal women with reduced bone mineral density. *Front Cell Infect Microbiol.* (2024) 14:1367325. 10.3389/fcimb.2024.1367325 38912210 PMC11190063

[172] Whitehead T Price N Drake H Cotta M. Catabolic pathway for the production of skatole and indoleacetic acid by the acetogen Clostridium drakei, Clostridium scatologenes, and swine manure. *Appl Environ Microbiol.* (2008) 74:1950–3. 10.1128/AEM.02458-07 18223109 PMC2268313

[173] Nie Q Sun Y Hu W Chen C Lin Q Nie S. Glucomannan promotes *Bacteroides* ovatus to improve intestinal barrier function and ameliorate insulin resistance. *Imeta.* (2024) 3:e163. 10.1002/imt2.163 38868507 PMC10989147

[174] Shen J Yang L You K Chen T Su Z Cui Z Indole-3-acetic acid alters intestinal microbiota and alleviates ankylosing spondylitis in mice. *Front Immunol.* (2022) 13:762580. 10.3389/fimmu.2022.762580 35185872 PMC8854167

[175] Sugama S Kakinuma Y. Noradrenaline as a key neurotransmitter in modulating microglial activation in stress response. *Neurochem Int.* (2021) 143:104943. 10.1016/j.neuint.2020.104943 33340593

[176] Del Toro-Barbosa M Hurtado-Romero A Garcia-Amezquita LE García-Cayuela T. Psychobiotics: Mechanisms of action, evaluation methods and effectiveness in applications with food products. *Nutrients.* (2020) 12:3896. 10.3390/nu12123896 33352789 PMC7767237

[177] Bo T Zhang X Wen J Deng K Qin X Wang D. The microbiota-gut-brain interaction in regulating host metabolic adaptation to cold in male Brandt’s voles (Lasiopodomys brandtii). *ISME J.* (2019) 13:3037–53. 10.1038/s41396-019-0492-y 31455805 PMC6863827

[178] Asano Y Hiramoto T Nishino R Aiba Y Kimura T Yoshihara K Critical role of gut microbiota in the production of biologically active, free catecholamines in the gut lumen of mice. *Am J Physiol Gastrointest Liver Physiol.* (2012) 303:G1288–95. 10.1152/ajpgi.00341.2012 23064760

[179] Mykel DG Mitchell BS Gilda AB. L-glutamine increases IGF-1 liver expression to prevent bone loss in sickle mice. *Blood.* (2019) 134:3561. 10.1182/blood-2019-128950

[180] Canalis E Agnusdei D. Insulin-like growth factors and their role in osteoporosis. *Calcif Tissue Int.* (1996) 58:133–4. 10.1007/BF02526877 8852566

[181] Chen C Tseng K Lai Y Chen Y Lin F Lin S. Overexpression of insulin-like growth factor 1 enhanced the osteogenic capability of aging bone marrow mesenchymal stem cells. *Theranostics.* (2017) 7:1598–611. 10.7150/thno.16637 28529639 PMC5436515

[182] Rosen CJ. Growth hormone, insulin-like growth factors, and the senescent skeleton: Ponce de Leon’s Fountain revisited? *J Cell Biochem.* (1994) 56:348–56. 10.1002/jcb.240560311 7876328

[183] Humam A Loh T Foo H Samsudin A Mustapha N Zulkifli I Effects of feeding different postbiotics produced by lactobacillus plantarum on growth performance, carcass yield, intestinal morphology, gut microbiota composition, immune status, and growth gene expression in broilers under heat stress. *Animals (Basel).* (2019) 9:644. 10.3390/ani9090644 31480791 PMC6770893

[184] Izuddin W Loh T Samsudin A Foo H Humam A Shazali N. Effects of postbiotic supplementation on growth performance, ruminal fermentation and microbial profile, blood metabolite and GHR, IGF-1 and MCT-1 gene expression in post-weaning lambs. *BMC Vet Res.* (2019) 15:315. 10.1186/s12917-019-2064-9 31477098 PMC6719353

[185] Zhang J Motyl K Irwin R MacDougald O Britton R McCabe L. Loss of bone and Wnt10b expression in male type 1 diabetic mice is blocked by the probiotic Lactobacillus reuteri. *Endocrinology.* (2015) 156:3169–82. 10.1210/EN.2015-1308 26135835 PMC4541610

[186] Matsushita M Fujita K Hayashi T Kayama H Motooka D Hase H Gut microbiota-derived short-chain fatty acids promote prostate cancer growth via IGF1 signaling. *Cancer Res.* (2021) 81:4014–26. 10.1158/0008-5472.CAN-20-4090 34039634

[187] Ding M Li B Chen H Liang D Ross R Stanton C Human breastmilk-derived Bifidobacterium longum subsp. infantis CCFM1269 regulates bone formation by the GH/IGF axis through PI3K/AKT pathway. *Gut Microbes.* (2024) 16:2290344. 10.1080/19490976.2023.2290344 38116652 PMC10761167

[188] Parada Venegas D De la Fuente MK Landskron G González MJ Quera R Dijkstra G Short chain fatty acids (SCFAs)-Mediated gut epithelial and immune regulation and its relevance for inflammatory bowel diseases. *Front Immunol.* (2019) 10:277. 10.3389/fimmu.2019.00277 30915065 PMC6421268

[189] Sonnenburg J Bäckhed F. Diet-microbiota interactions as moderators of human metabolism. *Nature.* (2016) 535:56–64. 10.1038/nature18846 27383980 PMC5991619

[190] Wang N Ma S Fu L. Gut microbiota dysbiosis as one cause of osteoporosis by impairing intestinal barrier function. *Calcif Tissue Int.* (2022) 110:225–35. 10.1007/s00223-021-00911-7 34480200

[191] Schepper J Collins F Rios-Arce N Kang H Schaefer L Gardinier J Involvement of the gut microbiota and barrier function in glucocorticoid-induced osteoporosis. *J Bone Miner Res.* (2020) 35:801–20. 10.1002/jbmr.3947 31886921

[192] Han H Kim J Choi Y Lee K Kwon T Kim S. Effect of Lactobacillus Fermentum as a probiotic agent on bone health in postmenopausal women. *J Bone Metab.* (2022) 29:225–33. 10.11005/jbm.2022.29.4.225 36529865 PMC9760773

[193] Harahap I Moszak M Czlapka-Matyasik M Skrypnik K Bogdański P Suliburska J. Effects of daily probiotic supplementation with Lactobacillus acidophilus on calcium status, bone metabolism biomarkers, and bone mineral density in postmenopausal women: A controlled and randomized clinical study. *Front Nutr.* (2024) 11:1401920. 10.3389/fnut.2024.1401920 39010860 PMC11247006

[194] Seyed Hameed A Rawat P Meng X Liu W. Biotransformation of dietary phytoestrogens by gut microbes: A review on bidirectional interaction between phytoestrogen metabolism and gut microbiota. *Biotechnol Adv.* (2020) 43:107576. 10.1016/j.biotechadv.2020.107576 32531317

[195] Ghimire S Cady N Lehman P Peterson S Shahi S Rashid F Dietary isoflavones alter gut microbiota and lipopolysaccharide biosynthesis to reduce inflammation. *Gut Microbes.* (2022) 14:2127446. 10.1080/19490976.2022.2127446 36179318 PMC9542810

[196] Baldi S Tristán Asensi M Pallecchi M Sofi F Bartolucci G Amedei A. Interplay between Lignans and gut microbiota: Nutritional, functional and methodological aspects. *Molecules.* (2023) 28:343. 10.3390/molecules28010343 36615537 PMC9822457

[197] Ma C Gao J Liang J Dai W Wang Z Xia M HDAC6 inactivates Runx2 promoter to block osteogenesis of bone marrow stromal cells in age-related bone loss of mice. *Stem Cell Res Ther.* (2021) 12:484. 10.1186/s13287-021-02545-w 34454588 PMC8403388

[198] Silva Y Bernardi A Frozza R. The role of short-chain fatty acids from gut microbiota in gut-brain communication. *Front Endocrinol (Lausanne).* (2020) 11:25. 10.3389/fendo.2020.00025 32082260 PMC7005631

[199] Singh N Gurav A Sivaprakasam S Brady E Padia R Shi H Activation of Gpr109a, receptor for niacin and the commensal metabolite butyrate, suppresses colonic inflammation and carcinogenesis. *Immunity.* (2014) 40:128–39. 10.1016/j.immuni.2013.12.007 24412617 PMC4305274

[200] Yano J Yu K Donaldson G Shastri G Ann P Ma L Indigenous bacteria from the gut microbiota regulate host serotonin biosynthesis. *Cell.* (2015) 161:264–76. 10.1016/j.cell.2015.02.047 25860609 PMC4393509

[201] Liu H Xiao H Lin S Zhou H Cheng Y Xie B Effect of gut hormones on bone metabolism and their possible mechanisms in the treatment of osteoporosis. *Front Pharmacol.* (2024) 15:1372399. 10.3389/fphar.2024.1372399 38725663 PMC11079205

[202] Harahap I Suliburska J. Probiotics and isoflavones as a promising therapeutic for calcium status and bone health: A narrative review. *Foods.* (2021) 10:2685. 10.3390/foods10112685 34828966 PMC8621960

[203] Wei P Liu M Chen Y Chen D. Systematic review of soy isoflavone supplements on osteoporosis in women. *Asian Pac J Trop Med.* (2012) 5:243–8. 10.1016/S1995-7645(12)60033-9 22305793

[204] Cano A García-Pérez M Tarín J. Isoflavones and cardiovascular disease. *Maturitas.* (2010) 67:219–26. 10.1016/j.maturitas.2010.07.015 20728290

[205] Pabich M Materska M. Biological effect of soy isoflavones in the prevention of civilization diseases. *Nutrients.* (2019) 11:1660. 10.3390/nu11071660 31330799 PMC6683102

[206] Clavel T Borrmann D Braune A Doré J Blaut M. Occurrence and activity of human intestinal bacteria involved in the conversion of dietary lignans. *Anaerobe.* (2006) 12:140–7. 10.1016/j.anaerobe.2005.11.002 16765860

[207] Setchell K Clerici C. Equol: History, chemistry, and formation. *J Nutr.* (2010) 140:1355S–62S. 10.3945/jn.109.119776 20519412 PMC2884333

[208] Mustafa S Mustafa S Ismail A Abas F Abd Manap M Ahmed Hamdi O Impact of prebiotics on equol production from soymilk isoflavones by two Bifidobacterium species. *Heliyon.* (2020) 6:e05298. 10.1016/j.heliyon.2020.e05298 33134584 PMC7586118

[209] Bravo D Peiroten A Alvarez I Landete J. Phytoestrogen metabolism by lactic acid bacteria: Enterolignan production by Lactobacillus salivarius and Lactobacillus gasseri strains. *J Funct Foods.* (2017) 37:373–8. 10.1016/j.jff.2017.08.015

[210] Ceccarelli I Bioletti L Peparini S Solomita E Ricci C Casini I Estrogens and phytoestrogens in body functions. *Neurosci Biobehav Rev.* (2022) 132:648–63. 10.1016/j.neubiorev.2021.12.007 34890602

[211] Gaya P Peirotén Á Medina M Landete JM. Isoflavone metabolism by a collection of lactic acid bacteria and bifidobacteria with biotechnological interest. *Int J Food Sci Nutr.* (2016) 67:117–24. 10.3109/09637486.2016.1144724 26878882

[212] Kiyama R. Estrogenic activity of coffee constituents. *Nutrients.* (2019) 11:1401. 10.3390/nu11061401 31234352 PMC6628280

[213] Langa S Peirotén Á Curiel JA de la Bastida AR Landete JM. Isoflavone metabolism by lactic acid bacteria and its application in the development of fermented soy food with beneficial effects on human health. *Foods.* (2023) 12:1293. 10.3390/foods12061293 36981219 PMC10048179

[214] Banc R Rusu M Filip L Popa D. The impact of ellagitannins and their metabolites through gut microbiome on the gut health and brain wellness within the gut-brain axis. *Foods.* (2023) 12:270. 10.3390/foods12020270 36673365 PMC9858309

[215] Mateş L Banc R Zaharie FA Rusu ME Popa DS. Mechanistic insights into the biological effects and antioxidant activity of walnut (Juglans regia L.) Ellagitannins: A systematic review. *Antioxidants (Basel).* (2024) 13:974. 10.3390/antiox13080974 39199220 PMC11351988

[216] García-Villalba R Tomás-Barberán F Iglesias-Aguirre C Giménez-Bastida J González-Sarrías A Selma M Ellagitannins, urolithins, and neuroprotection: Human evidence and the possible link to the gut microbiota. *Mol Aspects Med.* (2023) 89:101109. 10.1016/j.mam.2022.101109 35940941

[217] Li Y Wang F Li J Ivey K Wilkinson J Wang D Dietary lignans, plasma enterolactone levels, and metabolic risk in men: Exploring the role of the gut microbiome. *BMC Microbiol.* (2022) 22:82. 10.1186/s12866-022-02495-0 35350985 PMC8966171

[218] Clavel T Henderson G Engst W Doré J Blaut M. Phylogeny of human intestinal bacteria that activate the dietary lignan secoisolariciresinol diglucoside. *FEMS Microbiol Ecol.* (2006) 55:471–8. 10.1111/j.1574-6941.2005.00057.x 16466386

[219] Senizza A Rocchetti G Mosele J Patrone V Callegari M Morelli L Lignans and gut microbiota: An interplay revealing potential health implications. *Molecules.* (2020) 25:5709. 10.3390/molecules25235709 33287261 PMC7731202

[220] Chen C Dong B Wang Y Zhang Q Wang B Feng S The role of Bacillus acidophilus in osteoporosis and its roles in proliferation and differentiation. *J Clin Lab Anal.* (2020) 34:e23471. 10.1002/jcla.23471 32779308 PMC7676190

[221] Ghanemi A Melouane A Yoshioka M St-Amand J. Secreted protein acidic and rich in cysteine and bioenergetics: Extracellular matrix, adipocytes remodeling and skeletal muscle metabolism. *Int J Biochem Cell Biol.* (2019) 117:105627. 10.1016/j.biocel.2019.105627 31589923

[222] Rosset E Bradshaw AD. SPARC/osteonectin in mineralized tissue. *Matrix Biol.* (2016) 52-54:78–87. 10.1016/j.matbio.2016.02.001 26851678 PMC5327628

[223] Stevens J Miranda-Carboni G Singer M Brugger S Lyons K Lane T. Wnt10b deficiency results in age-dependent loss of bone mass and progressive reduction of mesenchymal progenitor cells. *J Bone Miner Res.* (2010) 25:2138–47. 10.1002/jbmr.118 20499361 PMC3153316

[224] Mirmohammadali S Gallant K Biruete A. Oh, My Gut! New insights on the role of the gastrointestinal tract and the gut microbiome in chronic kidney disease-mineral and bone disorder. *Curr Opin Nephrol Hypertens.* (2024) 33:226–30. 10.1097/MNH.0000000000000961 38088374 PMC11957419

[225] Lee H Lee J Kim S Jo S Min K. Probiotic Limosilactobacillus Reuteri (Lactobacillus Reuteri) extends the lifespan of drosophila melanogaster through Insulin/IGF-1 signaling. *Aging Dis.* (2023) 14:1407–24. 10.14336/AD.2023.0122 37163439 PMC10389828

[226] Kumar R Sharma A Gupta M Padwad Y Sharma R. Cell-free culture supernatant of probiotic Lactobacillus fermentum protects against H2O2-induced premature senescence by suppressing ROS-Akt-mTOR axis in murine preadipocytes. *Probiotics Antimicrob Proteins.* (2020) 12:563–76. 10.1007/s12602-019-09576-z 31332650

[227] Chen S Chen L Qi Y Xu J Ge Q Fan Y Bifidobacterium adolescentis regulates catalase activity and host metabolism and improves healthspan and lifespan in multiple species. *Nat Aging.* (2021) 1:991–1001. 10.1038/s43587-021-00129-0 37118342

[228] Madel M Halper J Ibáñez L Claire L Rouleau M Boutin A Specific targeting of inflammatory osteoclastogenesis by the probiotic yeast S. boulardii CNCM I-745 reduces bone loss in osteoporosis. *Elife.* (2023) 12:e82037. 10.7554/eLife.82037 36848406 PMC9977286

[229] Montazeri-Najafabady N Ghasemi Y Dabbaghmanesh M Talezadeh P Koohpeyma F Gholami A. Supportive role of probiotic strains in protecting rats from ovariectomy-induced cortical bone loss. *Probiotics Antimicrob Proteins.* (2019) 11:1145–54. 10.1007/s12602-018-9443-6 30014348

[230] Ai T Shang L Li B Li J Qin R. Konjac oligosaccharides alleviated ovariectomy-induced bone loss through gut microbiota modulation and Treg/Th17 regulation. *J Agric Food Chem.* (2024) 72:7969–79. 10.1021/acs.jafc.4c00281 38551374

[231] Nistal E Caminero A Herrán A Pérez-Andres J Vivas S Ruiz de Morales JM Study of duodenal bacterial communities by 16S rRNA gene analysis in adults with active celiac disease vs non-celiac disease controls. *J Appl Microbiol.* (2016) 120:1691–700. 10.1111/jam.13111 26913982

[232] Almonacid D Kraal L Ossandon F Budovskaya Y Cardenas J Bik E 16S rRNA gene sequencing and healthy reference ranges for 28 clinically relevant microbial taxa from the human gut microbiome. *PLoS One.* (2017) 12:e0176555. 10.1371/journal.pone.0176555 28467461 PMC5414997

[233] Mills S Stanton C Fitzgerald G Ross R. Enhancing the stress responses of probiotics for a lifestyle from gut to product and back again. *Microb Cell Fact.* (2011) 10:S19. 10.1186/1475-2859-10-S1-S19 21995734 PMC3231925

[234] Sagmeister T Gubensäk N Buhlheller C Grininger C Eder M Ðordić A The molecular architecture of Lactobacillus S-layer: Assembly and attachment to teichoic acids. *Proc Natl Acad Sci U S A.* (2024) 121:e2401686121. 10.1073/pnas.2401686121 38838019 PMC11181022

[235] Jansson P Curiac D Lazou Ahrén I Hansson F Martinsson Niskanen T Sjögren K Probiotic treatment using a mix of three Lactobacillus strains for lumbar spine bone loss in postmenopausal women: A randomised, double-blind, placebo-controlled, multicentre trial. *Lancet Rheumatol.* (2019) 1:e154–62. 10.1016/S2665-9913(19)30068-2 38229392

[236] Matar C Amiot J Savoie L Goulet J. The effect of milk fermentation by Lactobacillus helveticus on the release of peptides during in vitro digestion. *J Dairy Sci.* (1996) 79:971–9. 10.3168/jds.S0022-0302(96)76448-2 8827460

[237] Zhang X Li Y Zhang C Chi H Liu C Li A Postbiotics derived from Lactobacillus plantarum 1.0386 ameliorate lipopolysaccharide-induced tight junction injury via MicroRNA-200c-3p mediated activation of the MLCK-MLC pathway in Caco-2 cells. *Food Funct.* (2022) 13:11008–20. 10.1039/d2fo00001f 36040437

[238] Wei C Wu J Huang Y Wang X Li J. Lactobacillus plantarum improves LPS-induced Caco2 cell line intestinal barrier damage via cyclic AMP-PKA signaling. *PLoS One.* (2022) 17:e0267831. 10.1371/journal.pone.0267831 35639684 PMC9154120

[239] Karczewski J Troost F Konings I Dekker J Kleerebezem M Brummer R Regulation of human epithelial tight junction proteins by Lactobacillus plantarum in vivo and protective effects on the epithelial barrier. *Am J Physiol Gastrointest Liver Physiol.* (2010) 298:G851–9. 10.1152/ajpgi.00327.2009 20224007

[240] Guo M Liu H Yu Y Zhu X Xie H Wei C Lactobacillus rhamnosus GG ameliorates osteoporosis in ovariectomized rats by regulating the Th17/Treg balance and gut microbiota structure. *Gut Microbes.* (2023) 15:2190304. 10.1080/19490976.2023.2190304 36941563 PMC10038048

[241] Wang L Lu W Shi J Zhang H Xu X Gao B Anti-osteoporotic effects of tetramethylpyrazine via promoting osteogenic differentiation and inhibiting osteoclast formation. *Mol Med Rep.* (2017) 16:8307–14. 10.3892/mmr.2017.7610 28983593

[242] Jones M Martoni C Prakash S. Oral supplementation with probiotic L. reuteri NCIMB 30242 increases mean circulating 25-hydroxyvitamin D: A post hoc analysis of a randomized controlled trial. *J Clin Endocrinol Metab.* (2013) 98:2944–51. 10.1210/jc.2012-4262 23609838

[243] Li P Ji B Luo H Sundh D Lorentzon M Nielsen J. One-year supplementation with Lactobacillus reuteri ATCC PTA 6475 counteracts a degradation of gut microbiota in older women with low bone mineral density. *NPJ Biofilms Microbiomes.* (2022) 8:84. 10.1038/s41522-022-00348-2 36261538 PMC9581899

[244] Alvarez A Tap J Chambaud I Cools-Portier S Quinquis L Bourlioux P Safety and functional enrichment of gut microbiome in healthy subjects consuming a multi-strain fermented milk product: A randomised controlled trial. *Sci Rep.* (2020) 10:15974. 10.1038/s41598-020-72161-w 32994487 PMC7524715

[245] Rodrigues F Castro A Rodrigues V Fernandes S Fontes E de Oliveira T Yacon flour and Bifidobacterium longum modulate bone health in rats. *J Med Food.* (2012) 15:664–70. 10.1089/jmf.2011.0296 22510044

[246] Zhang J Liang X Tian X Zhao M Mu Y Yi H Bifidobacterium improves oestrogen-deficiency-induced osteoporosis in mice by modulating intestinal immunity. *Food Funct.* (2024) 15:1840–51. 10.1039/d3fo05212e 38273734

[247] Wallimann A Hildebrand M Groeger D Stanic B Akdis C Zeiter S An exopolysaccharide produced by Bifidobacterium longum 35624^®^ inhibits osteoclast formation via a TLR2-dependent mechanism. *Calcif Tissue Int.* (2021) 108:654–66. 10.1007/s00223-020-00790-4 33388801

[248] Zhao F Guo Z Kwok L Zhao Z Wang K Li Y Bifidobacterium lactis Probio-M8 improves bone metabolism in patients with postmenopausal osteoporosis, possibly by modulating the gut microbiota. *Eur J Nutr.* (2023) 62:965–76. 10.1007/s00394-022-03042-3 36334119

[249] Lu L Li J Liu L Wang C Xie Y Yu X Grape seed extract prevents oestrogen deficiency-induced bone loss by modulating the gut microbiota and metabolites. *Microb Biotechnol.* (2024) 17:e14485. 10.1111/1751-7915.14485 38850270 PMC11162104

[250] Silva Vde O Lobato R Andrade E de Macedo C Napimoga J Napimoga M β-Glucans (Saccharomyces cereviseae) reduce glucose levels and attenuate alveolar bone loss in diabetic rats with periodontal disease. *PLoS One.* (2015) 10:e0134742. 10.1371/journal.pone.0134742 26291983 PMC4546386

[251] Hong Y Jung E. Yeast hydrolysate and postmenopausal osteoporosis. *J Pers Med.* (2023) 13:322. 10.3390/jpm13020322 36836555 PMC9958730

[252] Ma Z Fu Q. Comparison of the therapeutic effects of yeast-incorporated gallium with those of inorganic gallium on ovariectomized osteopenic rats. *Biol Trace Elem Res.* (2010) 134:280–7. 10.1007/s12011-009-8472-0 19652924

[253] Ren Z Yang L Xue F Meng Q Wang K Wu X Yeast-incorporated gallium attenuates glucocorticoid-induced bone loss in rats by inhibition of bone resorption. *Biol Trace Elem Res.* (2013) 152:396–402. 10.1007/s12011-013-9634-7 23532566

[254] Arnaouteli S Bamford N Stanley-Wall N Kovács ÁT. Bacillus subtilis biofilm formation and social interactions. *Nat Rev Microbiol.* (2021) 19:600–14. 10.1038/s41579-021-00540-9 33824496

[255] Cutting S. Bacillus probiotics. *Food Microbiol.* (2011) 28:214–20. 10.1016/j.fm.2010.03.007 21315976

[256] Gholami A Dabbaghmanesh M Ghasemi Y Koohpeyma F Talezadeh P Montazeri-Najafabady N. The ameliorative role of specific probiotic combinations on bone loss in the ovariectomized rat model. *BMC Complement Med Ther.* (2022) 22:241. 10.1186/s12906-022-03713-y 36115982 PMC9482298

[257] Dar H Pal S Shukla P Mishra P Tomar G Chattopadhyay N Bacillus clausii inhibits bone loss by skewing Treg-Th17 cell equilibrium in postmenopausal osteoporotic mice model. *Nutrition.* (2018) 54:118–28. 10.1016/j.nut.2018.02.013 29793054

[258] Koh E Hwang I Lee H De Sotto R Lee J Lee Y Engineering probiotics to inhibit Clostridioides difficile infection by dynamic regulation of intestinal metabolism. *Nat Commun.* (2022) 13:3834. 10.1038/s41467-022-31334-z 35787625 PMC9253155

[259] Scott B Gutiérrez-Vázquez C Sanmarco L da Silva Pereira JA Li Z Plasencia A Self-tunable engineered yeast probiotics for the treatment of inflammatory bowel disease. *Nat Med.* (2021) 27:1212–22. 10.1038/s41591-021-01390-x 34183837

[260] Perreault M Means J Gerson E James M Cotton S Bergeron C The live biotherapeutic SYNB1353 decreases plasma methionine via directed degradation in animal models and healthy volunteers. *Cell Host Microbe.* (2024) 32:382–95.e10. 10.1016/j.chom.2024.01.005. 38309259

[261] Tan L Fu J Feng F Liu X Cui Z Li B Engineered probiotics biofilm enhances osseointegration via immunoregulation and anti-infection. *Sci Adv.* (2020) 6:eaba5723. 10.1126/sciadv.aba5723 33188012 PMC10763977

[262] Zheng L Wang H Zhong X Jia L Shi G Bai C Reprogramming tumor microenvironment with precise photothermal therapy by calreticulin nanobody-engineered probiotics. *Biomaterials.* (2025) 314:122809. 10.1016/j.biomaterials.2024.122809 39303415

[263] Fishbein S Evbuomwan E Dantas G. Conquering homocystinuria with engineered probiotics. *Cell Host Microbe.* (2024) 32:298–300. 10.1016/j.chom.2024.02.008 38484708 PMC12150346

[264] Yao M Xie J Du H McClements D Xiao H Li L. Progress in microencapsulation of probiotics: A review. *Compr Rev Food Sci Food Saf.* (2020) 19:857–74. 10.1111/1541-4337.12532 33325164

[265] Nakkarach A Foo H Song A Nitisinprasert S Withayagiat U. Promising discovery of beneficial *Escherichia coli* in the human gut. *3 Biotech.* (2020) 10:296. 10.1007/s13205-020-02289-z 32550113 PMC7283410

[266] Sonnenborn U. *Escherichia coli* strain Nissle 1917-from bench to bedside and back: History of a special *Escherichia coli* strain with probiotic properties. *FEMS Microbiol Lett.* (2016) 363:fnw212. 10.1093/femsle/fnw212 27619890

[267] Henker J Laass M Blokhin B Bolbot Y Maydannik V Elze M The probiotic *Escherichia coli* strain Nissle 1917 (EcN) stops acute diarrhoea in infants and toddlers. *Eur J Pediatr.* (2007) 166:311–8. 10.1007/s00431-007-0419-x 17287932 PMC1802727

[268] Kruis W Fric P Pokrotnieks J Lukás M Fixa B Kascák M Maintaining remission of ulcerative colitis with the probiotic *Escherichia coli* Nissle 1917 is as effective as with standard mesalazine. *Gut.* (2004) 53:1617–23. 10.1136/gut.2003.037747 15479682 PMC1774300

[269] Helmy Y Kassem I Rajashekara G. Immuno-modulatory effect of probiotic E. coli Nissle 1917 in polarized human colonic cells against Campylobacter jejuni infection. *Gut Microbes.* (2021) 13:1–16. 10.1080/19490976.2020.1857514 33382951 PMC7781529

[270] Sassone-Corsi M Nuccio S Liu H Hernandez D Vu C Takahashi A Microcins mediate competition among *Enterobacteriaceae* in the inflamed gut. *Nature.* (2016) 540:280–3. 10.1038/nature20557 27798599 PMC5145735

[271] Deane C. Secrete and protect. *Nat Chem Biol.* (2023) 19:537. 10.1038/s41589-023-01336-z 37117926

[272] Reister M Hoffmeier K Krezdorn N Rotter B Liang C Rund S Complete genome sequence of the gram-negative probiotic *Escherichia coli* strain Nissle 1917. *J Biotechnol.* (2014) 187:106–7. 10.1016/j.jbiotec.2014.07.442 25093936

[273] Zainuddin H Bai Y Mansell TJ. CRISPR-based curing and analysis of metabolic burden of cryptic plasmids in *Escherichia coli* Nissle 1917. *Eng Life Sci.* (2019) 19:478–85. 10.1002/elsc.201900003 32625025 PMC6999193

[274] Lynch J González-Prieto C Reeves A Bae S Powale U Godbole N Engineered *Escherichia coli* for the in situ secretion of therapeutic nanobodies in the gut. *Cell Host Microbe.* (2023) 31:634–49.e8. 10.1016/j.chom.2023.03.007. 37003258 PMC10101937

[275] Saleski T Chung M Carruthers D Khasbaatar A Kurabayashi K Lin X. Optimized gene expression from bacterial chromosome by high-throughput integration and screening. *Sci Adv.* (2021) 7:eabe1767. 10.1126/sciadv.abe1767 33579713 PMC7880599

[276] Seco E Fernández LÁ. Efficient markerless integration of genes in the chromosome of probiotic E. coli Nissle 1917 by bacterial conjugation. *Microb Biotechnol.* (2022) 15:1374–91. 10.1111/1751-7915.13967 34755474 PMC9049610

[277] Rottinghaus A Ferreiro A Fishbein S Dantas G Moon T. Genetically stable CRISPR-based kill switches for engineered microbes. *Nat Commun.* (2022) 13:672. 10.1038/s41467-022-28163-5 35115506 PMC8813983

[278] Chen H Lei P Ji H Yang Q Peng B Ma J Advances in *Escherichia coli* Nissle 1917 as a customizable drug delivery system for disease treatment and diagnosis strategies. *Mater Today Bio.* (2023) 18:100543. 10.1016/j.mtbio.2023.100543 36647536 PMC9840185

[279] Mimee M Nadeau P Hayward A Carim S Flanagan S Jerger L An ingestible bacterial-electronic system to monitor gastrointestinal health. *Science.* (2018) 360:915–8. 10.1126/science.aas9315 29798884 PMC6430580

[280] Praveschotinunt P Duraj-Thatte A Gelfat I Bahl F Chou D Joshi N. Engineered E. coli Nissle 1917 for the delivery of matrix-tethered therapeutic domains to the gut. *Nat Commun.* (2019) 10:5580. 10.1038/s41467-019-13336-6 31811125 PMC6898321

[281] Canale F Basso C Antonini G Perotti M Li N Sokolovska A Metabolic modulation of tumours with engineered bacteria for immunotherapy. *Nature.* (2021) 598:662–6. 10.1038/s41586-021-04003-2 34616044

[282] Leventhal D Sokolovska A Li N Plescia C Kolodziej S Gallant C Immunotherapy with engineered bacteria by targeting the STING pathway for anti-tumor immunity. *Nat Commun.* (2020) 11:2739. 10.1038/s41467-020-16602-0 32483165 PMC7264239

[283] Lan YJ Tan Y Cheng S Ting W Xue C Lin T Development of *Escherichia coli* Nissle 1917 derivative by CRISPR/Cas9 and application for gamma-aminobutyric acid (GABA) production in antibiotic-free system. *Biochem Eng J.* (2021) 168:107952. 10.1016/j.bej.2021.107952

[284] Cai H Zhou T Tang H Feng P Ali G Liu P Genetically encoded probiotic EcN 1917 alleviates alcohol-induced acute liver injury and restore gut microbiota homeostasis. *J Funct Foods.* (2021) 85:104661. 10.1016/j.jff.2021.104661

[285] Vockley J Sondheimer N Puurunen M Diaz G Ginevic I Grange D Efficacy and safety of a synthetic biotic for treatment of phenylketonuria: A phase 2 clinical trial. *Nat Metab.* (2023) 5:1685–90. 10.1038/s42255-023-00897-6 37770764

[286] Lubkowicz D Horvath N James M Cantarella P Renaud L Bergeron C An engineered bacterial therapeutic lowers urinary oxalate in preclinical models and in silico simulations of enteric hyperoxaluria. *Mol Syst Biol.* (2022) 18:e10539. 10.15252/msb.202110539 35253995 PMC8899768

[287] Khokhlova E Colom J Simon A Mazhar S García-Lainez G Llopis S Immunomodulatory and antioxidant properties of a novel potential probiotic Bacillus clausii CSI08. *Microorganisms.* (2023) 11:240. 10.3390/microorganisms11020240 36838205 PMC9962608

[288] Chollet L Heumel S Deruyter L Bouilloux F Delval L Robert V Faecalibacterium duncaniae as a novel next generation probiotic against influenza. *Front Immunol.* (2024) 15:1347676. 10.3389/fimmu.2024.1347676 38590519 PMC11000806

[289] Cani P Depommier C Derrien M Everard A de Vos W. Akkermansia muciniphila: Paradigm for next-generation beneficial microorganisms. *Nat Rev Gastroenterol Hepatol.* (2022) 19:625–37. 10.1038/s41575-022-00631-9 35641786

[290] Ross B. *Bacteroides* fragilis uses toxins for gut success. *Nat Microbiol.* (2024) 9:11–2. 10.1038/s41564-023-01569-7 38177303

[291] Yoo M Nguyen N Soucaille P. Trends in systems biology for the analysis and engineering of clostridium acetobutylicum metabolism. *Trends Microbiol.* (2020) 28:118–40. 10.1016/j.tim.2019.09.003 31627989

[292] Davey L Malkus P Villa M Dolat L Holmes Z Letourneau J A genetic system for Akkermansia muciniphila reveals a role for mucin foraging in gut colonization and host sterol biosynthesis gene expression. *Nat Microbiol.* (2023) 8:1450–67. 10.1038/s41564-023-01407-w 37337046 PMC11741908

[293] Ghelardi E Abreu Y Abreu AT Marzet CB Álvarez Calatayud G Perez M Current progress and future perspectives on the use of Bacillus clausii. *Microorganisms.* (2022) 10:1246. 10.3390/microorganisms10061246 35744764 PMC9230978

[294] Wexler H. *Bacteroides*: The good, the bad, and the nitty-gritty. *Clin Microbiol Rev.* (2007) 20:593–621. 10.1128/CMR.00008-07 17934076 PMC2176045

[295] Rios-Covian D Arboleya S Hernandez-Barranco A Alvarez-Buylla J Ruas-Madiedo P Gueimonde M Interactions between Bifidobacterium and *Bacteroides* species in cofermentations are affected by carbon sources, including exopolysaccharides produced by bifidobacteria. *Appl Environ Microbiol.* (2013) 79:7518–24. 10.1128/AEM.02545-13 24077708 PMC3837738

[296] Facchinetti F Jänne P Tiseo M. Chasing EGFR mutations in the plasma of patients With resected NSCLC: Lessons in the ADAURA Era. *J Thorac Oncol.* (2023) 18:1118–20. 10.1016/j.jtho.2023.06.019 37599043

[297] Lee J Tiffany C Mahan S Kellom M Rogers A Nguyen H High fat intake sustains sorbitol intolerance after antibiotic-mediated Clostridia depletion from the gut microbiota. *Cell.* (2024) 187:1191–205.e15. 10.1016/j.cell.2024.01.029. 38366592 PMC11023689

[298] Kadowaki R Tanno H Maeno S Endo A. Spore-forming properties and enhanced oxygen tolerance of butyrate-producing Anaerostipes spp. *Anaerobe.* (2023) 82:102752. 10.1016/j.anaerobe.2023.102752 37301503

[299] Lyu Z Yuan G Zhang Y Zhang F Liu Y Li Y Anaerostipes caccae CML199 enhances bone development and counteracts aging-induced bone loss through the butyrate-driven gut-bone axis: The chicken model. *Microbiome.* (2024) 12:215. 10.1186/s40168-024-01920-y 39438898 PMC11495078

[300] Hesser L Puente A Arnold J Ionescu E Mirmira A Talasani N A synbiotic of Anaerostipes caccae and lactulose prevents and treats food allergy in mice. *Cell Host Microbe.* (2024) 32:1163–76.e6. 10.1016/j.chom.2024.05.019. 38906158 PMC11239278

[301] Chen S Wang H. An engineered cas-transposon system for programmable and site-directed DNA transpositions. *CRISPR J.* (2019) 2:376–94. 10.1089/crispr.2019.0030 31742433 PMC6919251

[302] Wiles T Norton J Russell C Dalley B Fischer K Mulvey M. Combining quantitative genetic footprinting and trait enrichment analysis to identify fitness determinants of a bacterial pathogen. *PLoS Genet.* (2013) 9:e1003716. 10.1371/journal.pgen.1003716 23990803 PMC3749937

[303] Zhang J Hong W Guo L Wang Y Wang Y. Enhancing plasmid transformation efficiency and enabling CRISPR-Cas9/Cpf1-based genome editing in Clostridium tyrobutyricum. *Biotechnol Bioeng.* (2020) 117:2911–7. 10.1002/bit.27435 32437010

[304] Zhou X Wang X Luo H Wang Y Wang Y Tu T Exploiting heterologous and endogenous CRISPR-Cas systems for genome editing in the probiotic Clostridium butyricum. *Biotechnol Bioeng.* (2021) 118:2448–59. 10.1002/bit.27753 33719068

[305] Tajkarimi M Wexler HM. CRISPR-Cas systems in Bacteroides fragilis, an important pathobiont in the human gut microbiome. *Front Microbiol.* (2017) 8:2234. 10.3389/fmicb.2017.02234 29218031 PMC5704556

[306] Zafar H Saier M. Gut *Bacteroides* species in health and disease. *Gut Microbes.* (2021) 13:1–20. 10.1080/19490976.2020.1848158 33535896 PMC7872030

[307] Zhang J Huang Y Yoon J Kemmitt J Wright C Schneider K Primary human colonic mucosal barrier crosstalk with super oxygen-sensitive Faecalibacterium prausnitzii in continuous culture. *Med.* (2021) 2:74–98.e9. 10.1016/j.medj.2020.07.001. 33511375 PMC7839961

[308] Shepherd E DeLoache W Pruss K Whitaker W Sonnenburg J. An exclusive metabolic niche enables strain engraftment in the gut microbiota. *Nature.* (2018) 557:434–8. 10.1038/s41586-018-0092-4 29743671 PMC6126907

[309] Kothari D Patel S Kim S. Probiotic supplements might not be universally-effective and safe: A review. *Biomed Pharmacother.* (2019) 111:537–47. 10.1016/j.biopha.2018.12.104 30597307

[310] Rao S Yu S Tetangco E Yan Y. Probiotics can cause D-Lactic acidosis and brain fogginess: Reply to Quigley et al. *Clin Transl Gastroenterol.* (2018) 9:207. 10.1038/s41424-018-0077-5 30449886 PMC6240559

[311] Suez J Zmora N Zilberman-Schapira G Mor U Dori-Bachash M Bashiardes S Post-antibiotic gut mucosal microbiome reconstitution is impaired by probiotics and improved by autologous FMT. *Cell.* (2018) 174:1406–23.e16. 10.1016/j.cell.2018.08.047. 30193113

[312] Larsson D Flach C. Antibiotic resistance in the environment. *Nat Rev Microbiol.* (2022) 20:257–69. 10.1038/s41579-021-00649-x 34737424 PMC8567979

[313] Lerner A Matthias T Aminov R. Potential effects of horizontal gene exchange in the human gut. *Front Immunol.* (2017) 8:1630. 10.3389/fimmu.2017.01630 29230215 PMC5711824

[314] Charbonneau M Isabella V Li N Kurtz C. Developing a new class of engineered live bacterial therapeutics to treat human diseases. *Nat Commun.* (2020) 11:1738. 10.1038/s41467-020-15508-1 32269218 PMC7142098

[315] Hehemann J Correc G Barbeyron T Helbert W Czjzek M Michel G. Transfer of carbohydrate-active enzymes from marine bacteria to Japanese gut microbiota. *Nature.* (2010) 464:908–12. 10.1038/nature08937 20376150

[316] Eliaz I. The failure of probiotics-and the strategy of microbiome synergy. *Integr Med (Encinitas).* (2020) 19:8–10. PMC7572142 33132772

[317] Wang X Lin S Wang L Cao Z Zhang M Zhang Y Versatility of bacterial outer membrane vesicles in regulating intestinal homeostasis. *Sci Adv.* (2023) 9:eade5079. 10.1126/sciadv.ade5079 36921043 PMC10017049

[318] Aguilera L Toloza L Giménez R Odena A Oliveira E Aguilar J Proteomic analysis of outer membrane vesicles from the probiotic strain *Escherichia coli* Nissle 1917. *Proteomics.* (2014) 14:222–9. 10.1002/pmic.201300328 24307187

[319] Bishop D Work E. An extracellular glycolipid produced by *Escherichia coli* grown under lysine-limiting conditions. *Biochem J.* (1965) 96:567–76. 10.1042/bj0960567 4953781 PMC1207076

[320] Dorward D Garon CF. DNA is packaged within membrane-derived vesicles of gram-negative but not gram-positive bacteria. *Appl Environ Microbiol.* (1990) 56:1960–2. 10.1128/aem.56.6.1960-1962.1990 16348232 PMC184538

[321] Turnbull L Toyofuku M Hynen A Kurosawa M Pessi G Petty N Explosive cell lysis as a mechanism for the biogenesis of bacterial membrane vesicles and biofilms. *Nat Commun.* (2016) 7:11220. 10.1038/ncomms11220 27075392 PMC4834629

[322] Brown L Wolf J Prados-Rosales R Casadevall A. Through the wall: Extracellular vesicles in Gram-positive bacteria, mycobacteria and fungi. *Nat Rev Microbiol.* (2015) 13:620–30. 10.1038/nrmicro3480 26324094 PMC4860279

[323] Zhang Y Li Y Lu P Dai G Chen X Rui Y. The modulatory effect and implication of gut microbiota on osteoporosis: From the perspective of brain-gut-bone axis. *Food Funct.* (2021) 12:5703–18. 10.1039/d0fo03468a 34048514

[324] Sharon G Garg N Debelius J Knight R Dorrestein P Mazmanian S. Specialized metabolites from the microbiome in health and disease. *Cell Metab.* (2014) 20:719–30. 10.1016/j.cmet.2014.10.016 25440054 PMC4337795

[325] Zou Y Huang B Cao L Deng Y Su J. Tailored mesoporous inorganic biomaterials: Assembly, functionalization, and drug delivery engineering. *Adv Mater.* (2021) 33:e2005215. 10.1002/adma.202005215 33251635

[326] Mora-Raimundo P Lozano D Manzano M Vallet-Regí M. Nanoparticles to knockdown osteoporosis-related gene and promote osteogenic marker expression for osteoporosis treatment. *ACS Nano.* (2019) 13:5451–64. 10.1021/acsnano.9b00241 31071265 PMC6588271

[327] Tan J Wang C Wang D Jiang H Qiao Y Zhang D Tailoring time-varying alkaline microenvironment on titanium for sequential anti-infection and osseointegration. *Chem Eng J.* (2022) 431:133940. 10.1016/j.cej.2021.133940

[328] Shuai C Xu Y Feng P Wang G Xiang S Peng S Antibacterial polymer scaffold based on mesoporous bioactive glass loaded with in situ grown silver. *Chem Eng J.* (2019) 374:304–15. 10.1016/j.cej.2019.03.273

[329] Zhang Z Zhang X Wang C Teng W Xing H Wang F Enhancement of motor functional recovery using immunomodulatory extracellular vesicles-loaded injectable thermosensitive hydrogel post spinal cord injury. *Chem Eng J.* (2022) 433:134465. 10.1016/j.cej.2021.134465

[330] Wang T Mo L Ou J Fang Q Wu H Wu Y *Proteus mirabilis* vesicles induce mitochondrial apoptosis by regulating miR96-5p/Abca1 to inhibit osteoclastogenesis and bone loss. *Front Immunol.* (2022) 13:833040. 10.3389/fimmu.2022.833040 35242136 PMC8885728

[331] Liu H Song P Zhang H Zhou F Ji N Wang M Synthetic biology-based bacterial extracellular vesicles displaying BMP-2 and CXCR4 to ameliorate osteoporosis. *J Extracell Vesicles.* (2024) 13:e12429. 10.1002/jev2.12429 38576241 PMC10995478

[332] Liu H Wu Y Wang Y Wang S Ji N Wang M Bone-targeted engineered bacterial extracellular vesicles delivering miRNA to treat osteoporosis. *Composites B Eng.* (2023) 267:111047. 10.1016/j.compositesb.2023.111047

[333] Xie X Cheng P Hu L Zhou W Zhang D Knoedler S Bone-targeting engineered small extracellular vesicles carrying anti-miR-6359-CGGGAGC prevent valproic acid-induced bone loss. *Signal Transduct Target Ther.* (2024) 9:24. 10.1038/s41392-023-01726-8 38246920 PMC10800355

[334] Lin Y Wu J Gu W Huang Y Tong Z Huang L Exosome-liposome hybrid nanoparticles deliver CRISPR/Cas9 system in MSCs. *Adv Sci (Weinh).* (2018) 5:1700611. 10.1002/advs.201700611 29721412 PMC5908366

[335] Piffoux M Silva A Wilhelm C Gazeau F Tareste D. Modification of extracellular vesicles by fusion with liposomes for the design of personalized biogenic drug delivery systems. *ACS Nano.* (2018) 12:6830–42. 10.1021/acsnano.8b02053 29975503

[336] Hu Y Li X Zhang Q Gu Z Luo Y Guo J Exosome-guided bone targeted delivery of Antagomir-188 as an anabolic therapy for bone loss. *Bioact Mater.* (2021) 6:2905–13. 10.1016/j.bioactmat.2021.02.014 33718671 PMC7917458

[337] Chen G Bai Y Li Z Wang F Fan X Zhou X. Bacterial extracellular vesicle-coated multi-antigenic nanovaccines protect against drug-resistant Staphylococcus aureus infection by modulating antigen processing and presentation pathways. *Theranostics.* (2020) 10:7131–49. 10.7150/thno.44564 32641983 PMC7330855

[338] Chen Q Huang G Wu W Wang J Hu J Mao J A Hybrid eukaryotic-prokaryotic nanoplatform with photothermal modality for enhanced antitumor vaccination. *Adv Mater.* (2020) 32:e1908185. 10.1002/adma.201908185 32108390

[339] Alvarez-Erviti L Seow Y Yin H Betts C Lakhal S Wood M. Delivery of siRNA to the mouse brain by systemic injection of targeted exosomes. *Nat Biotechnol.* (2011) 29:341–5. 10.1038/nbt.1807 21423189

[340] Zha Y Li Y Lin T Chen J Zhang S Wang J. Progenitor cell-derived exosomes endowed with VEGF plasmids enhance osteogenic induction and vascular remodeling in large segmental bone defects. *Theranostics.* (2021) 11:397–409. 10.7150/thno.50741 33391482 PMC7681080

[341] Aqil F Munagala R Jeyabalan J Agrawal A Kyakulaga A Wilcher S Milk exosomes - Natural nanoparticles for siRNA delivery. *Cancer Lett.* (2019) 449:186–95. 10.1016/j.canlet.2019.02.011 30771430

[342] Wu P Zhang B Ocansey D Xu W Qian H. Extracellular vesicles: A bright star of nanomedicine. *Biomaterials.* (2021) 269:120467. 10.1016/j.biomaterials.2020.120467 33189359

[343] Yi K Rong Y Huang L Tang X Zhang Q Wang W Aptamer-exosomes for tumor theranostics. *ACS Sens.* (2021) 6:1418–29. 10.1021/acssensors.0c02237 33755415

[344] Gao X Ran N Dong X Zuo B Yang R Zhou Q Anchor peptide captures, targets, and loads exosomes of diverse origins for diagnostics and therapy. *Sci Transl Med.* (2018) 10:eaat0195. 10.1126/scitranslmed.aat0195 29875202

[345] Kalluri R LeBleu V. The biology, function, and biomedical applications of exosomes. *Science.* (2020) 367:eaau6977. 10.1126/science.aau6977 32029601 PMC7717626

[346] Wang J Li W Lu Z Zhang L Hu Y Li Q The use of RGD-engineered exosomes for enhanced targeting ability and synergistic therapy toward angiogenesis. *Nanoscale.* (2017) 9:15598–605. 10.1039/c7nr04425a 28990632

[347] Nakase I Futaki S. Combined treatment with a pH-sensitive fusogenic peptide and cationic lipids achieves enhanced cytosolic delivery of exosomes. *Sci Rep.* (2015) 5:10112. 10.1038/srep10112 26011176 PMC4443764

[348] Sawada S Sato Y Kawasaki R Yasuoka J Mizuta R Sasaki Y Nanogel hybrid assembly for exosome intracellular delivery: Effects on endocytosis and fusion by exosome surface polymer engineering. *Biomater Sci.* (2020) 8:619–30. 10.1039/c9bm01232j 31833484

[349] Yang L Han D Zhan Q Li X Shan P Hu Y Blood TfR+ exosomes separated by a pH-responsive method deliver chemotherapeutics for tumor therapy. *Theranostics.* (2019) 9:7680–96. 10.7150/thno.37220 31695794 PMC6831460

[350] Pan C Li J Hou W Lin S Wang L Pang Y Polymerization-mediated multifunctionalization of living cells for enhanced cell-based therapy. *Adv Mater.* (2021) 33:e2007379. 10.1002/adma.202007379 33629757

[351] Peng B Yang Y Wu Z Tan R Pham T Yeo E Red blood cell extracellular vesicles deliver therapeutic siRNAs to skeletal muscles for treatment of cancer cachexia. *Mol Ther.* (2023) 31:1418–36. 10.1016/j.ymthe.2023.03.036 37016578 PMC10188904

[352] Huang C Kang M Shirazi S Lu Y Cooper L Gajendrareddy P 3D Encapsulation and tethering of functionally engineered extracellular vesicles to hydrogels. *Acta Biomater.* (2021) 126:199–210. 10.1016/j.actbio.2021.03.030 33741538 PMC8096714

[353] Ma L Ke W Liao Z Feng X Lei J Wang K Small extracellular vesicles with nanomorphology memory promote osteogenesis. *Bioact Mater.* (2022) 17:425–38. 10.1016/j.bioactmat.2022.01.008 35386457 PMC8964989

[354] Deng J Wang X Zhang W Sun L Han X Tang H Versatile Hypoxic extracellular vesicles laden in an injectable and bioactive hydrogel for accelerated bone regeneration. *Adv Funct Mater.* (2023) 33:2211664. 10.1002/adfm.202211664

[355] Chen Q Bai H Wu W Huang G Li Y Wu M Bioengineering bacterial vesicle-coated polymeric nanomedicine for enhanced cancer immunotherapy and metastasis prevention. *Nano Lett.* (2020) 20:11–21. 10.1021/acs.nanolett.9b02182 31858807

[356] Zhuang Q Xu J Deng D Chao T Li J Zhang R Bacteria-derived membrane vesicles to advance targeted photothermal tumor ablation. *Biomaterials.* (2021) 268:120550. 10.1016/j.biomaterials.2020.120550 33278684

[357] Li M Zhou H Yang C Wu Y Zhou X Liu H Bacterial outer membrane vesicles as a platform for biomedical applications: An update. *J Control Release.* (2020) 323:253–68. 10.1016/j.jconrel.2020.04.031 32333919

[358] Grozdanov L Raasch C Schulze J Sonnenborn U Gottschalk G Hacker J Analysis of the genome structure of the nonpathogenic probiotic *Escherichia coli* strain Nissle 1917. *J Bacteriol.* (2004) 186:5432–41. 10.1128/JB.186.16.5432-5441.2004 15292145 PMC490877

[359] Park K Svennerholm K Crescitelli R Lässer C Gribonika I Lötvall J. Synthetic bacterial vesicles combined with tumour extracellular vesicles as cancer immunotherapy. *J Extracell Vesicles.* (2021) 10:e12120. 10.1002/jev2.12120 34262675 PMC8254025

[360] Cheng K Kang Q Zhao X. Biogenic nanoparticles as immunomodulator for tumor treatment. *Wiley Interdiscip Rev Nanomed Nanobiotechnol.* (2020) 12:e1646. 10.1002/wnan.1646 32464709

[361] Qing S Lyu C Zhu L Pan C Wang S Li F Biomineralized bacterial outer membrane vesicles potentiate safe and efficient tumor microenvironment reprogramming for anticancer therapy. *Adv Mater.* (2020) 32:e2002085. 10.1002/adma.202002085 33015871

[362] Klimentová J Stulík J. Methods of isolation and purification of outer membrane vesicles from gram-negative bacteria. *Microbiol Res.* (2015) 170:1–9. 10.1016/j.micres.2014.09.006 25458555

[363] Wang Z Shi Y Liu F Wang H Liu X Sun R Distributed mobile ultraviolet light sources driven by ambient mechanical stimuli. *Nano Energy.* (2020) 74:104910. 10.1016/j.nanoen.2020.104910 32373446 PMC7198214

[364] Bitto N Zavan L Johnston E Stinear T Hill A Kaparakis-Liaskos M. Considerations for the analysis of bacterial membrane vesicles: Methods of vesicle production and quantification can influence biological and experimental outcomes. *Microbiol Spectr.* (2021) 9:e0127321. 10.1128/Spectrum.01273-21 34937167 PMC8694105

[365] Gandham S Su X Wood J Nocera A Alli S Milane L Technologies and standardization in research on extracellular vesicles. *Trends Biotechnol.* (2020) 38:1066–98. 10.1016/j.tibtech.2020.05.012 32564882 PMC7302792

[366] Cheng K Zhao R Li Y Qi Y Wang Y Zhang Y Bioengineered bacteria-derived outer membrane vesicles as a versatile antigen display platform for tumor vaccination via plug-and-display technology. *Nat Commun.* (2041) 12:2041. 10.1038/s41467-021-22308-8 33824314 PMC8024398

[367] Li Y Zhao R Cheng K Zhang K Wang Y Zhang Y Bacterial outer membrane vesicles presenting programmed death 1 for improved cancer immunotherapy via immune activation and checkpoint inhibition. *ACS Nano.* (2020) 14:16698–711. 10.1021/acsnano.0c03776 33232124

[368] Thomas S Madaan T Kamble N Siddiqui N Pauletti G Kotagiri N. Engineered bacteria enhance immunotherapy and targeted therapy through stromal remodeling of tumors. *Adv Healthc Mater.* (2022) 11:e2101487. 10.1002/adhm.202101487 34738725 PMC8770579

[369] Gerritzen M Maas R van den Ijssel J van Keulen L Martens D Wijffels R High dissolved oxygen tension triggers outer membrane vesicle formation by *Neisseria meningitidis*. *Microb Cell Fact.* (2018) 17:157. 10.1186/s12934-018-1007-7 30285743 PMC6171317

[370] Hong J Dauros-Singorenko P Whitcombe A Payne L Blenkiron C Phillips A Analysis of the *Escherichia coli* extracellular vesicle proteome identifies markers of purity and culture conditions. *J Extracell Vesicles.* (2019) 8:1632099. 10.1080/20013078.2019.1632099 31275533 PMC6598517

[371] Dauros Singorenko P Chang V Whitcombe A Simonov D Hong J Phillips A Isolation of membrane vesicles from prokaryotes: A technical and biological comparison reveals heterogeneity. *J Extracell Vesicles.* (2017) 6:1324731. 10.1080/20013078.2017.1324731 28717421 PMC5505020

